# Tunable unconventional kagome superconductivity in charge ordered RbV_3_Sb_5_ and KV_3_Sb_5_

**DOI:** 10.1038/s41467-022-35718-z

**Published:** 2023-01-11

**Authors:** Z. Guguchia, C. Mielke, D. Das, R. Gupta, J.-X. Yin, H. Liu, Q. Yin, M. H. Christensen, Z. Tu, C. Gong, N. Shumiya, Md Shafayat Hossain, Ts. Gamsakhurdashvili, M. Elender, Pengcheng Dai, A. Amato, Y. Shi, H. C. Lei, R. M. Fernandes, M. Z. Hasan, H. Luetkens, R. Khasanov

**Affiliations:** 1 Laboratory for Muon Spin Spectroscopy, Paul Scherrer Institute, CH-5232 Villigen PSI, Switzerland; 2 Department of Physics, Southern University of Science and Technology, Shenzhen, Guangdong 518055 China; 3 Beijing National Laboratory for Condensed Matter Physics and Institute of Physics, Chinese Academy of Sciences, 100190 Beijing, China; 4 University of Chinese Academy of Sciences, 100049 Beijing, China; 5 Department of Physics and Beijing Key Laboratory of Opto-electronic Functional Materials and Micro-nano Devices, Renmin University of China, 100872 Beijing, China; 6 Niels Bohr Institute, University of Copenhagen, Copenhagen, 2100 Denmark; 7 Laboratory for Topological Quantum Matter and Advanced Spectroscopy (B7), Department of Physics, Princeton University, Princeton, NJ 08544 USA; 8 Department of Physics and Astronomy, Rice University, Houston, TX 77005 USA; 9 School of Physics and Astronomy, University of Minnesota, Minneapolis, MN 55455 USA; 10 Princeton Institute for the Science and Technology of Materials, Princeton University, Princeton, NJ 08540 USA; 11 Materials Sciences Division, Lawrence Berkeley National Laboratory, Berkeley, CA 94720 USA; 12 Quantum Science Center, Oak Ridge, TN, 37831 USA

**Keywords:** Superconducting properties and materials, Electronic properties and materials

## Abstract

Unconventional superconductors often feature competing orders, small superfluid density, and nodal electronic pairing. While unusual superconductivity has been proposed in the kagome metals *A*V_3_Sb_5_, key spectroscopic evidence has remained elusive. Here we utilize pressure-tuned and ultra-low temperature muon spin spectroscopy to uncover the unconventional nature of superconductivity in RbV_3_Sb_5_ and KV_3_Sb_5_. At ambient pressure, we observed time-reversal symmetry breaking charge order below ${T}_{{{{{{{{\rm{1}}}}}}}}}^{*}\simeq$ 110 K in RbV_3_Sb_5_ with an additional transition at ${T}_{{{{{{{{\rm{2}}}}}}}}}^{*}\simeq$ 50 K. Remarkably, the superconducting state displays a nodal energy gap and a reduced superfluid density, which can be attributed to the competition with the charge order. Upon applying pressure, the charge-order transitions are suppressed, the superfluid density increases, and the superconducting state progressively evolves from nodal to nodeless. Once optimal superconductivity is achieved, we find a superconducting pairing state that is not only fully gapped, but also spontaneously breaks time-reversal symmetry. Our results point to unprecedented tunable nodal kagome superconductivity competing with time-reversal symmetry-breaking charge order and offer unique insights into the nature of the pairing state.

## Abstract (web summary)

The nature of the superconductivity in the kagome metals AV_3_Sb_5_ (A = K, Rb, Cs) remains under debate. Here, using muon spin spectroscopy, the authors find that the superconductivity in RbV_3_Sb_5_ and KV_3_Sb_5_ evolves from nodal to nodeless with increasing pressure and the superconducting state breaks time-reversal symmetry after suppression of the charge order.

## Introduction

**Fig. 1:**
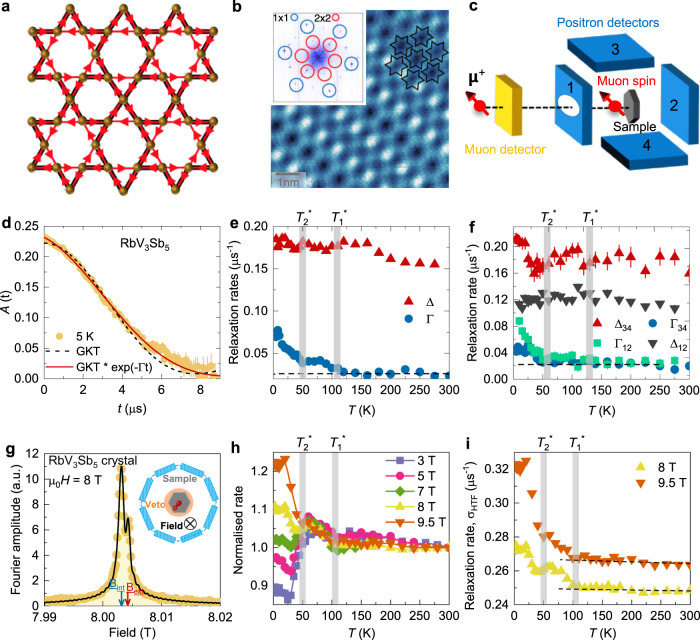
Time-reversal symmetry-breaking charge order in RbV_3_Sb_5_. **a** Schematic example of an orbital current state (red arrows) in the kagome lattice. **b** Scanning tunneling microscopy of the Sb surface showing 2 × 2 charge order as illustrated by black lines. The inset is the Fourier transform of this image, displaying 1 × 1 lattice Bragg peaks (blue circles) and 2 × 2 charge-order peaks (red circles). The latter have different intensities, suggesting a chirality of the charge order. **c** A schematic overview of the experimental set-up (see the “Methods” section). **d** The ZF *μ*SR time spectra for the polycrystalline sample of RbV_3_Sb_5_, obtained at *T* = 5 K. The dashed and solid curves represent fits using the Gaussian Kubo–Toyabe (GKT) function without (black) and with (red) a multiplied exponential $\exp (-\Gamma t)$ term, respectively. Error bars are the standard error of the mean (s.e.m.) in about 10^6^ events. The temperature dependences of the relaxation rates Δ and Γ, which can be related to the nuclear and electronic contributions respectively, are shown in a wide temperature range for the polycrystalline (**e**) and the single crystal samples (**f**) of RbV_3_Sb_5_. **f** presents Γ obtained from two sets of detectors. The error bars represent the standard deviation of the fit parameters. **g** Fourier transform of the *μ*SR asymmetry spectra for the single crystal of RbV_3_Sb_5_ at 3 K in the presence of an applied field of *μ*_0_*H* = 8T. The black solid line is a two-component signal fit. The peaks marked by the arrows denote the external and internal fields, determined as the mean values of the field distribution from the silver sample holder (mostly) and from the sample, respectively. Inset shows the schematic high-field *μ*SR experimental set-up (see the “Methods” section). **h** The temperature dependence of the high transverse field muon spin relaxation rate *σ*_HTF_ for the single crystal of RbV_3_Sb_5_, normalized to the value at 300 K, measured under different *c*-axis magnetic fields. **i** The temperature dependence of the relaxation rate, measured under magnetic field values of *μ*_0_*H* = 8 and 9.5 T.

Due to their inherent geometric frustration and unique band structure, kagome materials^1^ represent an excellent platform for discovering, classifying and understanding correlated electronic phases of quantum matter^2–6^. The novel family of kagome metals *A*V_3_Sb_5_ (*A* = K, Rb, Cs)^7–11^ exhibit an array of interesting effects such as giant anomalous Hall conductivity^12,13^, charge order^11,14–23^, orbital order^24^, and possible unconventional superconductivity^7,8,10^. An important feature of the charge order, which onsets at temperatures *T*_co_ ~ 100 K at ambient pressure, is the breaking of time-reversal symmetry, as reported by scanning tunneling microscopy (STM) measurements^11,14,15,17^ in (K,Rb,Cs)V_3_Sb_5_, by muon spin relaxation (*μ*SR) in KV_3_Sb_5_^25^ and CsV_3_Sb_5_^26,27^, and by Kerr effect measurements in CsV_3_Sb_5_^28^. This implies that the charge-ordered state displays not only bond distortions, but also orbital current loops (see Fig. 1a)^29–32^.

Similarly to charge order, superconductivity, with transition temperature *T*_*c*_ ~ 1 K at ambient pressure, was also reported to display intriguing features, such as multiple gaps in (K,Cs)V_3_Sb_5_^33–36^, diminished superfluid density in KV_3_Sb_5_^25^, and double-dome structures in the pressure phase diagrams of all three compounds^37–39^. However, no consensus on the superconducting gap structure has been reached yet^25,33–35,40–43^, partly due to the challenges of performing spectroscopic studies under extreme conditions including ultra low temperatures and large pressures. Moreover, the role of the unconventional charge order in the emergence of these unusual superconducting features remains unclear, since the former onsets at a much higher temperature than the latter. In this regard, the sensitivity of both *T*_*c*_ and *T*_co_ on the applied pressure^37–39^ offers a rare setting to study the interplay between these two orders with a disorder-free tuning knob.

Here, we tackle these issues by employing zero-field and high-field muon spin relaxation experiments to directly probe the interplay between charge order and superconductivity across the temperature-pressure phase diagram of RbV_3_Sb_5_. This allows us to assess not only the time-reversal symmetry-breaking nature of these two states, but also the evolution of the low-energy superconducting excitations as *T*_co_ is suppressed and *T*_c_ is enhanced. The latter measurements unearth a remarkable pressure induced crossover from nodal pairing, when superconductivity coexists with charge order, to nodeless pairing, when optimal superconductivity is achieved near the critical pressure for charge order. They also reveal distinct relationships between *T*_*c*_ and the superfluid density in these two regimes. The same behaviors are also observed in KV_3_Sb_5_, attesting to the robustness of our conclusions for the understanding of the pairing mechanism in the *A*V_3_Sb_5_ family. We discuss different scenarios for the symmetries of both the superconductivity and charge order states that may account for the unusual nodal-to-nodeless transition.

## Results and discussion

### Probing spontaneous time-reversal symmetry breaking

Scanning tunneling microscopy observes 2 × 2 charge order in RbV_3_Sb_5_ (Fig. 1b and ref. 14) with an unusual magnetic field response^14^, suggestive of time-reversal symmetry-breaking charge order. To directly probe signatures of time-reversal symmetry-breaking, we carried out zero field (ZF) *μ*SR experiments (see Fig. 1c) on both single crystal and polycrystalline samples of RbV_3_Sb_5_ above and below *T*_co_. The ZF-*μ*SR spectra (see Fig. 1d) were fitted using the Gaussian Kubo–Toyabe (GKT) depolarization function^44^ multiplied with an exponential decay function^25^:

1 $${P}_{{{{{{\rm{ZF}}}}}}}^{{{{{{{{\rm{GKT}}}}}}}}}(t)=\left(\frac{1}{3}+\frac{2}{3}(1-{\Delta }^{2}{t}^{2})\exp \left[-\frac{{\Delta }^{2}{t}^{2}}{2}\right]\right)\exp (-\Gamma t)$$

here, Δ/*γ*_*μ*_ is the width of the local field distribution due to the nuclear moments and *γ*_*μ*_ = 0.085 *μ*s^−1^ G^−1^ is the muon gyromagnetic ratio. A GKT shape is expected due to the presence of the dense system of nuclear moments with large values of nuclear spins (*I* = 3/2 for ^39^K, *I* = 7/2 for ^51^V, and *I* = 5/2 for ^121^Sb) in KV_3_Sb_5_ and a high natural abundance. The exponential relaxation rate Γ is mostly sensitive to the temperature dependence of the electronic contribution to the muon spin relaxation, as we discussed previously in our work that reported time-reversal symmetry-breaking in KV_3_Sb_5_^25^. Because the zero-field relaxation is decoupled by the application of a small external magnetic field applied in a direction longitudinal to the muon spin polarization, *B*_LF_ = 50 G (see Supplementary Note 4 and Supplementary Fig. 4), the relaxation is, therefore, due to spontaneous fields that are static at the microsecond timescale^45^. In Fig. 1e, the temperature dependence of the Gaussian and exponential relaxation rates Δ and Γ for the polycrystalline sample of RbV_3_Sb_5_ are shown over a broad temperature range. The main observation is the two-step increase of the relaxation rate Γ, consisting of a noticeable enhancement at ${T}_{{{{{{{{\rm{1}}}}}}}}}^{*}\simeq$ 110 K, which corresponds approximately to the charge-order transition temperature *T*_co_, and a stronger increase below ${T}_{{{{{{{{\rm{2}}}}}}}}}^{*}\simeq$ 50 K. To substantiate this result, data from the single crystals are presented in Fig. 1f. The data from the up–down (34) and forward–backward (12) sets of detectors not only confirm the increase of Γ, but also shed more light into the origin of the two-step behavior. In particular, while Γ_34_ is enhanced mostly below ${T}_{{{{{{{{\rm{2}}}}}}}}}^{*}\simeq$ 50 K, Γ_12_ also features a mild initial increase right below ${T}_{{{{{{{{\rm{1}}}}}}}}}^{*}\simeq$ 110 K. Since the enhanced electronic relaxation rate below ${T}_{{{{{{{{\rm{1}}}}}}}}}^{*}$ is seen mostly in Γ_12_, it indicates that the local field at the muon site lies mostly within the *ab*-plane of the crystal. Below ${T}_{{{{{{{{\rm{2}}}}}}}}}^{*}$, the internal field also acquires an out-of-plane component, as manifested by the enhancement of both Γ_12_ and Γ_34_. The increase of the electronic contribution to the internal field width is also accompanied by maxima and minima in the temperature dependence of the nuclear contribution to the internal field width Δ/*γ*_*μ*_, particularly for the up–down set of detectors (Fig. 1e, f).

The increase in the exponential relaxation of RbV_3_Sb_5_ between ${T}_{{{{{{{{\rm{1}}}}}}}}}^{*}$ and 2 K is about 0.05 *μ*s^−1^, which can be interpreted as a characteristic field strength Γ_12_/*γ*_*μ*_ ≃ 0.6 G. While these ZF-*μ*SR results are consistent with the onset of time-reversal symmetry-breaking at *T*_co_, high-field *μ*SR experiments, illustrated in the inset of Fig. 1g, are essential to confirm this effect. As we discussed previously^25^, the onset of charge order might also alter the electric field gradient experienced by the nuclei and correspondingly the magnetic dipolar coupling of the muon to the nuclei. This can induce a change in the nuclear dipole contribution to the zero-field *μ*SR signal. In a high magnetic field, the direction of the applied field defines the quantization axis for the nuclear moments, so that the effect of the charge order on the electric field gradient at the nuclear sites is irrelevant. Figure 1g shows the probability distribution of the magnetic field measured at 3 K for the single-crystal samples of RbV_3_Sb_5_ in the presence of a *c*-axis magnetic field of 8 T (see “Methods” for the details of the analysis, see also Supplementary Note 5 and Supplementary Fig. 5). The contribution from the internal field is clearly identified. Figure 1h shows the corresponding temperature-dependent relaxation rate *σ*_HTF_ for different values of the external *c*-axis field. For 3 T, it displays a non-monotonic behavior, staying nearly constant across ${T}_{{{{{{{{\rm{1}}}}}}}}}^{*}$ and then decreasing to a minimum before increasing again at low temperatures. Upon increasing the external field, the relaxation rate not only shows an increase right at ${T}_{{{{{{{{\rm{1}}}}}}}}}^{*}\simeq$ 110 K, but its temperature dependence below ${T}_{{{{{{{{\rm{2}}}}}}}}}^{*}$ is reversed from being reduced to being enhanced upon lowering the temperature. Thus, as shown in Fig. 1, the relaxation rate extracted from the high-field *μ*SR data shows a qualitatively similar two-step increase as the ZF data at the same characteristic temperatures ${T}_{{{{{{{{\rm{1}}}}}}}}}^{*}\simeq$ 110 K and ${T}_{{{{{{{{\rm{2}}}}}}}}}^{*}\simeq$ 50 K—although the features are more pronounced at high fields. Because the temperature dependence of the nuclear contribution to the relaxation cannot be changed by an external field, we conclude that the two-step increase in the relaxation rate is driven by the electronic/magnetic contribution.

**Fig. 2:**
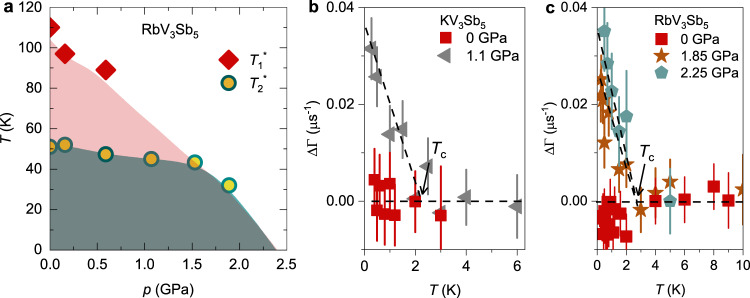
Time-reversal symmetry-breaking charge order and superconductivity in (K,Rb)V_3_Sb_5_ under pressure. **a** The pressure dependences of the transition temperatures ${T}_{{{{{{{{\rm{1}}}}}}}}}^{*}$ and ${T}_{{{{{{{{\rm{2}}}}}}}}}^{*}$. Temperature dependence of the absolute change of the electronic relaxation rate ΔΓ = Γ(*T*) − Γ(*T* > *T*_c_) for KV_3_Sb_5_ (**b**) and RbV_3_Sb_5_ (**c**) in the temperature range across *T*_c_, measured at ambient pressure and above the critical pressure at which *T*_c_ is maximum. The error bars represent the standard deviation of the fit parameters.

Therefore, the combination of ZF-*μ*SR and high-field *μ*SR results on RbV_3_Sb_5_ provide direct evidence for time-reversal symmetry-breaking below the onset of charge order, which approximately coincides with ${T}_{{{{{{{{\rm{1}}}}}}}}}^{*}\simeq$ 110 K. It is important to note that the entire sample volume experiences an increase in the relaxation rate, indicating the bulk nature of the transition below ${T}_{{{{{{{{\rm{1}}}}}}}}}^{*}$. As we previously discussed for KV_3_Sb_5_^25^, one plausible scenario to explain this effect is that loop currents along the kagome bonds are generated by a complex charge order parameter^16,22,23^. Within this framework, muons can couple to the fields generated by these loop currents via their spin, resulting in an enhanced internal field width sensed by the muon ensemble (see also Supplementary Information). The lower-temperature increase of the relaxation rate at ${T}_{{{{{{{{\rm{2}}}}}}}}}^{*}\simeq$ 50 K is suggestive of another ordered state that modifies such loop currents. An obvious candidate is a secondary charge-ordered state onsetting at ${T}_{{{{{{{{\rm{2}}}}}}}}}^{*}$. Indeed, experimentally, it has been reported that RbV_3_Sb_5_ and CsV_3_Sb_5_ kagome metals may display two charge-order transitions^19–21^. Theoretically, different charge-order instabilities have been found in close proximity^18^. Because time-reversal symmetry is already broken at ${T}_{{{{{{{{\rm{1}}}}}}}}}^{*}$, it is not possible to distinguish, with our *μ*SR data, whether this secondary charge-order state would break time-reversal symmetry on its own, or whether it is a more standard type of bond-charge-order. The two transitions in the relaxation rate observed in RbV_3_Sb_5_ is different from KV_3_Sb_5_, where only one transition is observed. This suggests the presence of only one type of TRSB charge order in KV_3_Sb_5_, while multiple nearby magnetic-charge order instabilities seem to be present in RbV_3_Sb_5_. Therefore, the TRSB effects in RbV_3_Sb_5_ are clearly different that those in KV_3_Sb_5_^25^. Furthermore, we followed these two transitions in RbV_3_Sb_5_ as a function of pressure (see the details in the Supplementary Note 7 and the Supplementary Fig. 7). As shown in Fig. 2a, both ${T}_{{{{{{{{\rm{1}}}}}}}}}^{*}$ and ${T}_{{{{{{{{\rm{2}}}}}}}}}^{*}$ are suppressed by hydrostatic pressure. More specifically, the two-step charge-order transition becomes a single time-reversal symmetry-breaking charge-order transition at ~1.5 GPa, above which ${T}_{{{{{{{{\rm{1}}}}}}}}}^{*}$ = ${T}_{{{{{{{{\rm{2}}}}}}}}}^{*}$ shows a faster suppression (see the “Methods” section for details).

**Fig. 3:**
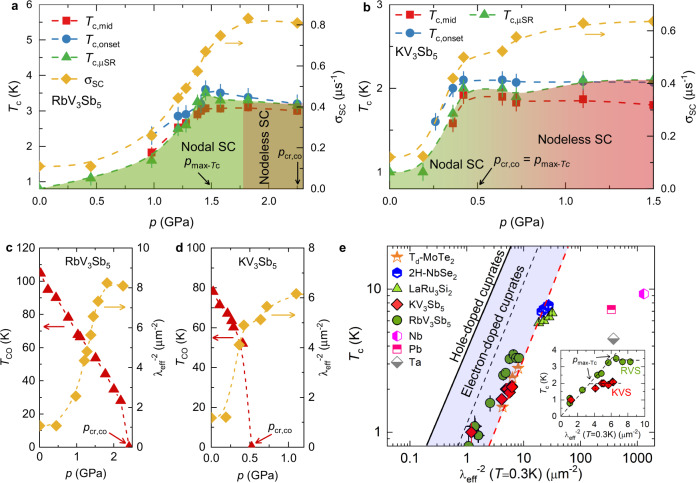
Coupled charge order and nodal superconductivity in kagome lattice. Pressure dependence of the superconducting transition temperature (left axis) and of the base-*T* value of *σ*_sc_ (right axis) for the polycrystalline samples of RbV_3_Sb_5_ (**a**) and KV_3_Sb_5_ (**b**). Here, *T*_c,ons_ and *T*_c,mid_ were obtained from AC measurements and *T*_c,μSR_, from *μ*SR. Pressure dependence of ${\lambda }_{{{{{{{{\rm{eff}}}}}}}}}^{-2}$ and charge order temperature *T*_co_ (ref. 38) for RbV_3_Sb_5_ (**c**) and KV_3_Sb_5_ (ref. 39) (**d**). The arrows mark the critical pressure *p*_cr,co_ at which charge order is suppressed and the pressure ${p}_{\max -{{{{{{{\rm{Tc}}}}}}}}}$ at which *T*_c_ reaches its maximum value. **e** Plot of *T*_c_ versus ${\lambda }_{{{{{{{{\rm{eff}}}}}}}}}^{-2}(0)$ in logarithmic scale obtained from our *μ*SR experiments in KV_3_Sb_5_ and RbV_3_Sb_5_. Inset shows the plot in a linear scale. The dashed red line represents the relationship obtained for the kagome superconductor LaRu_3_Si_2_ as well as for the layered transition metal dichalcogenide superconductors *T*_*d*_-MoTe_2_ and 2H-NbSe_2_^51,52^. The relationship observed for cuprates is also shown^50^, as are the points for various conventional superconductors. The error bars represent the standard deviation of the fit parameters.

The same ZF-*μ*SR analysis can also be employed to probe whether there is time-reversal symmetry-breaking inside the superconducting state. Because charge order already breaks time-reversal symmetry at *T*_co_ ≫ *T*_*c*_, it is necessary to suppress *T*_co_, which can be accomplished with pressure. Pressure of 1.85 GPa allows to enter the optimal *T*_c_ region of the phase diagram (see Fig. 3a) in RbV_3_Sb_5_ at which only a single time-reversal symmetry-breaking charge order transition is observed (see Fig. 2a). The maximum pressure we could apply (2.25 GPa) is enough to completely suppress the charge-order in RbV_3_Sb_5_. The pressure value of *p* > 0.5 GPa is large enough to assess the pure superconducting state of the related compound KV_3_Sb_5_. In Fig. 2b, we show the behavior of the internal field width Γ, extracted from the ZF-*μ*SR data, across the superconducting transition of KV_3_Sb_5_ measured both at ambient pressure (red, where charge-order is present) and at 1.1 GPa (gray, where charge-order is absent). While at ambient pressure Γ is little affected by superconductivity, at the higher pressure there is a significant enhancement of Γ, comparable to what has been observed in superconductors that are believed to spontaneously break time-reversal symmetry, such as SrRu_2_O_4_^46^. The similar enhancement of Γ below *T*_c_ ~ 3 K is observed for RbV_3_Sb_5_ at *p* = 1.85 and 2.25 GPa, as shown in Fig. 2c. This provides strong evidence for time-reversal symmetry-breaking superconducting states in KV_3_Sb_5_ and RbV_3_Sb_5_, indicative of an unconventional pairing state.

### Superfluid density as a function of pressure

**Fig. 4:**
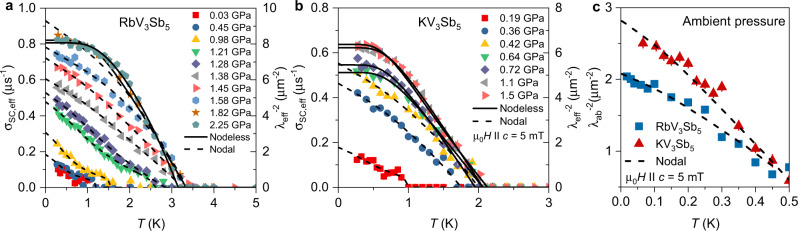
Tunable nodal kagome superconductivity. The temperature dependence of the superconducting muon spin depolarization rates *σ*_sc_ for RbV_3_Sb_5_ (**a**) and KV_3_Sb_5_ (**b**), measured in an applied magnetic field of *μ*_0_*H* = 5 mT at ambient and various applied hydrostatic pressures. The error bars represent the standard deviations of the fit parameters. The solid (dashed) lines correspond to a fit using a model with nodeless (nodal) two-gap superconductivity. **c** The inverse squared penetration depth ${\lambda }_{ab}^{-2}$ for the single crystals of KV_3_Sb_5_ and RbV_3_Sb_5_ as a function of temperature at ambient pressure.

An additional property of the superconducting state that can be directly measured with *μ*SR is the superfluid density. This is accomplished by extracting the second moment of the field distribution from the muon spin depolarization rate *σ*_sc_, which is related to the superconducting magnetic penetration depth *λ* as $< \Delta {B}^{2} > \propto {\sigma }_{sc}^{2}\propto {\lambda }^{-4}$ (see the “Methods” section). Because *λ*^−2^ is proportional to the superfluid density, so is *σ*_*sc*_. Figures 3 and 4 summarize the pressure and temperature dependences of *σ*_sc_ (measured in an applied magnetic field of *μ*_0_*H* = 5 mT) in the superconducting states of RbV_3_Sb_5_ and KV_3_Sb_5_. As the temperature is decreased below *T*_c_, the depolarization rate *σ*_sc_ starts to increase from zero due to the formation of the flux-line lattice (see Fig. 4a). As the pressure is increased, not only *T*_c_ (as determined from AC susceptibility and *μ*SR experiments), but also the low-temperature value of *σ*_sc_ (measured at the baseline of 0.25 K) show a substantial increase for both compounds, as shown in Fig. 3a, b. In both cases, *T*_c,ons_ first quickly reaches a maximum at a characteristic pressure ${p}_{\max -{{{{{{{\rm{Tc}}}}}}}}}$, namely, 3.5 K at ${p}_{\max -{{{{{{{\rm{Tc}}}}}}}}}\simeq$ 1.5 GPa for the Rb compound and ≃2.1 K at ${p}_{\max -{{{{{{{\rm{Tc}}}}}}}}}\simeq$ 0.5 GPa for the K compound. Beyond those pressure values, the transition temperature remains nearly unchanged. The superfluid density *σ*_sc_ (0.25 K) also increases significantly from its ambient-pressure value upon approaching ${p}_{\max -{{{{{{{\rm{Tc}}}}}}}}}$, by a factor of approximately 7 for the Rb compound and 5 for the K system. In both cases, *σ*_sc_ (0.25 K) continues increasing beyond ${p}_{\max -{{{{{{{\rm{Tc}}}}}}}}}$, although at a lower rate that may indicate approach to saturation.

These behaviors are consistent with competition between charge order and superconductivity. Indeed, as shown in Fig. 3c, d, the increase in the superfluid density is correlated with the decrease in the charge ordering temperature *T*_co_. More specifically, the pressure values ${p}_{\max -{{{{{{{\rm{Tc}}}}}}}}}$ for which *T*_*c*_ is maximum are close to the critical pressures *p*_cr,co_ beyond which charge order is completely suppressed. In fact, as displayed in Fig. 3b, *p*_cr,co_ essentially coincides with ${p}_{\max -{{{{{{{\rm{Tc}}}}}}}}}$ for KV_3_Sb_5_. Since both superconductivity and charge order occurs in the entire sample volume, there is no volume wise competition of these orders in real space. They rather compete for the same electronic states in reciprocal space. In this case, competition with charge order could naturally account for the suppression of the superfluid density towards the low-pressure region of the phase diagram, where *T*_co_ is the largest. Since charge order partially gaps the Fermi surface, as recently seen by quantum oscillation^47^ and ARPES^17,34^ studies, the electronic states available for the superconducting state are suppressed, thus decreasing the superfluid density^48,49^.

Next, it is important to note some differences in the temperature-pressure phase diagrams of RbV_3_Sb_5_ (Fig. 3a) and KV_3_Sb_5_ (Fig. 3b). For RbV_3_Sb_5_, *T*_c_ as well as *σ*_sc_ shows a smooth increase with increasing pressure until it reaches a maximum value at ${p}_{\max -{{{{{{{\rm{Tc}}}}}}}}}\simeq$ 1.5 GPa. In contrast, in KV_3_Sb_5_, both *T*_c_ and *σ*_sc_ show a sharp increase upon approaching ${p}_{\max -{{{{{{{\rm{Tc}}}}}}}}}\simeq$ 0.5 GPa. This suggests that the transition from the charge ordered state to optimal superconducting state is more likely first-order in KV_3_Sb_5_ and second-order in RbV_3_Sb_5_. Another difference is that for the RbV_3_Sb_5_, ${p}_{\max -{{{{{{{\rm{Tc}}}}}}}}}\simeq$ 1.5 GPa is lower than the critical pressure *p*_cr_ ≃ 2.2 GPa^38^, while in KV_3_Sb_5_ these two pressures coincide ${p}_{\max -{{{{{{{\rm{Tc}}}}}}}}}\simeq {p}_{{{{{{{{\rm{cr}}}}}}}}}\simeq$ 0.5 GPa.

Having extracted *σ*_sc_, we can directly obtain the magnetic penetration depth *λ* (see “Methods”). For polycrystalline samples, this gives an effective penetration depth *λ*_eff_, whereas for single crystals, it gives the in-plane *λ*_*ab*_. It is instructive to plot the low-temperature penetration depth not as a function of pressure, but as a function of *T*_c_^50^. As shown in Fig. 3e, the ratio ${T}_{{{{{{{{\rm{c}}}}}}}}}/{\lambda }_{{{{{{{{\rm{eff}}}}}}}}}^{-2}$ for unpressurized RbV_3_Sb_5_ is ~0.7 K *μ*m^−2^, similar to the one previously reported for KV_3_Sb_5_^25^. This ratio value is significantly larger from that of conventional BCS superconductors, indicative of a much smaller superfluid density. Moreover, we also find an unusual relationship between ${\lambda }_{{{{{{{{\rm{eff}}}}}}}}}^{-2}$ and *T*_c_ in these two kagome superconductors, which is not expected for conventional superconductivity. This is presented in the inset of Fig. 3e: below ${p}_{\max -{{{{{{{\rm{Tc}}}}}}}}}$, the superfluid density (which is proportional to ${\lambda }_{{{{{{{{\rm{eff}}}}}}}}}^{-2}$) depends linearly on *T*_c_, whereas above ${p}_{\max -{{{{{{{\rm{Tc}}}}}}}}}$, *T*_*c*_ barely changes for increasing ${\lambda }_{{{{{{{{\rm{eff}}}}}}}}}^{-2}$. Historically, a linear increase of *T*_c_ with ${\lambda }_{{{{{{{{\rm{eff}}}}}}}}}^{-2}$ has been observed only in the underdoped region of the phase diagram of unconventional superconductors. Deviations from linear behavior were previously found in an optimally doped cuprate^51^, in some Fe-based superconductors^52^, and in the charge-ordered superconductor 2H-NbSe_2_ under pressure^51^. Therefore, in RbV_3_Sb_5_ and KV_3_Sb_5_, it is tempting to attribute this deviation to the suppression of the competing charge ordered state by the applied pressure. More broadly, these two different dependences of *T*_c_ with ${\lambda }_{{{{{{{{\rm{eff}}}}}}}}}^{-2}$ indicate superconducting states with different properties below and above ${p}_{\max -{{{{{{{\rm{Tc}}}}}}}}}$.

To further probe this scenario, we quantitatively analyze the temperature dependence of the penetration depth *λ*(*T*)^53^ for both compounds as a function of pressure, see Fig. 4a, b. Quite generally, upon decreasing the temperature towards zero, a power-law dependence of ${\lambda }_{{{{{{{{\rm{eff}}}}}}}}}^{-2}(T)$ is indicative of the presence of nodal quasiparticles, whereas an exponential saturation-like behavior is a signature of a fully gapped spectrum. The low-*T* behavior of ${\lambda }_{ab}^{-2}(T)$ for single crystals of RbV_3_Sb_5_ and KV_3_Sb_5_, measured down to 18 mK and shown in Fig. 4c, displays a linear-in-*T* behavior, consistent with the presence of gap nodes. Quantitatively, the curve is well described by a phenomenological two-gap model, where one of the gaps has nodes and the other does not (see “Methods”). Such a linear-in-*T* increase of ${\lambda }_{{{{{{{{\rm{eff}}}}}}}}}^{-2}(T)$ upon approaching *T* = 0 is also seen in polycrystalline samples for pressures up to ${p}_{\max -{{{{{{{\rm{Tc}}}}}}}}}$. In the case of RbV_3_Sb_5_ (Fig. 4a), for two pressure values available above ${p}_{\max -{{{{{{{\rm{Tc}}}}}}}}}\approx 1.5$ GPa, the penetration depth curves seem to be better fitted by a model with a nodeless gap (see the Supplementary Note 13 and Supplementary Figs. 11 and 13). This conclusion is supported by a ${{\chi }_{r}}^{2}$-comparison, revealing a value of reduced ${{\chi }_{r}}^{2}$ at *p* = 1.85 GPa and *p* = 2.25 GPa for the nodal gap model that is higher by factor of ~3.9 than the one for the nodeless gap model. The analysis clearly shows that at least ten points below 2 K do not follow a linear temperature dependence. This is also clear in the case of KV_3_Sb_5_ (Fig. 4b): above ${p}_{\max -{{{{{{{\rm{Tc}}}}}}}}}\approx 0.5$ GPa, ${\lambda }_{{{{{{{{\rm{eff}}}}}}}}}^{-2}(T)$ displays a saturation-like behavior that is well captured quantitatively by a model with a nodeless gap (see Supplementary Figs. 12 and 13). Since ${p}_{\max -{{{{{{{\rm{Tc}}}}}}}}}$ is close to *p*_co,cr_, especially for the K compound, these results show that charge order strongly influences the superconducting gap structure in (Rb,K)V_3_Sb_5_, inducing nodes in an otherwise fully gapped pairing state. To the best of our knowledge this is the first direct experimental demonstration of a plausible pressure-induced change in the superconducting gap structure from nodal to nodeless in these kagome superconductors.

One possible explanation for these results is on the changes that the emergence of charge order causes on the Fermi surface. First-principle calculations on AV_3_Sb_5_ compounds indicate the existence of multiple Fermi pockets in the absence of charge order^47^. The simplest fully-gapped pairing state is an s-wave one consisting of different nodeless gaps (with potentially different signs) around each pocket. The onset of long-range charge order further breaks up these pockets into additional smaller pockets. Depending on the relative sign between the original gaps and on the details of the reconstructed Fermi pockets, accidental nodes could emerge. Such a scenario was proposed in the case of competing *s*^+−^-wave superconductivity and spin-density wave in iron-pnictide superconductors^54^.

The main drawback of this scenario is that it does not account for the time-reversal symmetry-breaking of the “pure" superconducting state. In this regard, a fully gapped pairing state that also breaks time-reversal symmetry is the chiral ${d}_{{x}^{2}-{y}^{2}}+i{d}_{xy}$ state^55,56^. As long as the charge ordered state preserves the point-group symmetry of the disordered state, the chiral pairing symmetry is expected to be retained below *T*_co_, suggesting a nodeless superconducting state. However, if the charge-ordered state breaks the threefold rotational symmetry of the lattice, as proposed experimentally^57^ and theoretically^18,22^ for certain AV_3_Sb_5_ compounds, a nodal gap is stabilized for a sufficiently large charge order parameter, as we show in the Supplementary Fig. 14 (see the Supplementary Note 14). In this case, the nodal-to-nodeless transition does not coincide with the full suppression of charge order, unless the transition from the charge-ordered superconducting state to the “pure" superconducting state is first-order. We note that the same conclusions would also apply for the triplet chiral *p*_*x*_ + *ip*_*y*_ state. As it was mentioned above, our phase diagrams suggest that the transition from the charge ordered superconducting state to optimal superconducting state in KV_3_Sb_5_ is more likely first-order and second-order in RbV_3_Sb_5_. Within the framework of our theoretical model, we expect the crossover from nodal to nodeless pairing to start at a lower pressure than *p*_cr_ in RbV_3_Sb_5_ and at *p*_cr_ in KV_3_Sb_5_. This is in excellent qualitative agreement with the experimental results.

In conclusion, our results provide direct evidence for unconventional superconductivity in (Rb,K)V_3_Sb_5_, by combining the observations of nodal superconducting pairing and a small superfluid density at ambient pressure, which in turn displays an unconventional dependence on the superconducting critical temperature. Moreover, we find that the hydrostatic pressure induces a change from a nodal superconducting gap structure at low pressure to a nodeless, time-reversal symmetry-breaking superconducting gap structure at high pressure. The crossover from nodal to nodeless pairing is correlated with the establishment of the optimal superconducting region of the phase diagram, which corresponds to full suppression of charge order in KV_3_Sb_5_ and partial suppression of charge order in RbV_3_Sb_5_. Our results point to the rich interplay and accessible tunability between nodal unconventional superconductivity and time-reversal symmetry-breaking charge orders in the correlated kagome lattice, offering new insights into the microscopic mechanisms involved in both orders.

## Methods

### Experimental details

ZF and transverse field (TF) *μ*SR experiments at ambient pressure on the single crystalline and polycrystalline samples of RbV_3_Sb_5_ and KV_3_Sb_5_ were performed on the GPS instrument and high-field HAL-9500 instrument, equipped with BlueFors vacuum-loaded cryogen-free dilution refrigerator (DR), at the Swiss Muon Source (S*μ*S) at the Paul Scherrer Institut, in Villigen, Switzerland. *μ*SR experiments under pressure were performed at the *μ*E1 beamline of the Paul Scherrer Institute (Villigen, Switzerland using the instrument GPD, where an intense high-energy (*p*_*μ*_ = 100 MeV c^−1^) beam of muons is implanted in the sample through the pressure cell. The ^4^He cryostats equipped with the ^3^He insets (base temperature ≃ 0.25 K) were used. A mosaic of several crystals stacked on top of each other was used for these measurements. The magnetic field was applied both in-plane (along the *ab*-plane) and out-of-plane (along the crystallographic *c*-axis). A schematic overview of the experimental setup for zero-field and low transverse field measurements is shown in Fig. 1c. The crystal was mounted such that the *c*-axis of it is parallel to the muon beam. Using the ”spin rotator” at the *π*M3 beamline, muon spin was rotated (from its natural orientation, which is antiparallel to the momentum of the muon) by 44.5(3)° with respect to the *c*-axis of the crystal. So, the sample orientation is fixed but the muon spin was rotated. The rotation angel can be precisely estimated to be 44.5(3)° by measurements in weak magnetic field, applied transverse to the muon spin polarization. The sample was surrounded by four detectors: Forward (1), Backward (2), Up (3) and Down (4). A schematic overview of the experimental setup for high-field *μ*SR instrument is shown in the inset of Fig. 1g. The muon spin forms 90° with respect to the *c*-axis of the crystal. The sample was surrounded by 2 times 8 positron detectors, arranged in rings. The specimen was mounted in a He gas-flow cryostat with the largest face perpendicular to the muon beam direction, along which the external field was applied. Zero field and high transverse field *μ*SR data analysis on single crystals of RbV_3_Sb_5_ were performed using both the so-called asymmetry and single-histogram modes^45^.

### Sample growth

Single crystals of RbV_3_Sb_5_ were synthesized by Rb ingot (purity 99.9%), V powder (purity 99.9%), and Sb grains (purity 99.999%) using the self-flux method^10^. Magnetization experiments reveal two characteristic temperatures of ${T}_{{{{{{{{\rm{1}}}}}}}}}^{*}\simeq$ 110 K and ${T}_{{{{{{{{\rm{2}}}}}}}}}^{*}\simeq$ 50 K (see Supplementary Note 3 and Supplementary Fig. 3).

### *μ*SR experiment

In a *μ*SR experiment^58^, nearly 100% spin-polarized muons *μ*^+^ are implanted into the sample one at a time. The positively charged *μ*^+^ thermalize at interstitial lattice sites, where they act as magnetic microprobes. In a magnetic material, the muon spin precesses in the local field *B*_*μ*_ at the muon site with the Larmor frequency *ν*_*μ*_ = *γ*_*μ*_/(2*π*)*B*_*μ*_ (muon gyromagnetic ratio *γ*_*μ*_/(2*π*) = 135.5 MHz T^−1^). Using the *μ*SR technique, important length scales of superconductors can be measured, namely, the magnetic penetration depth *λ* and the coherence length *ξ*. If a type II superconductor is cooled below *T*_c_ in an applied magnetic field ranging between the lower (*H*_*c*1_) and the upper (*H*_*c*2_) critical fields, a vortex lattice is formed which in general is incommensurate with the crystal lattice, with vortex cores separated by much larger distances than those of the crystallographic unit cell. Because the implanted muons stop at given crystallographic sites, they will randomly probe the field distribution of the vortex lattice. Such measurements need to be performed in a field applied perpendicular to the initial muon spin polarization (so-called TF configuration).

The magnetic penetration depth *λ*(*T*) is related to the superconducting muon spin depolarization rate *σ*_SC_(*T*) in the presence of a triangular (or hexagonal) vortex lattice by the equation^58^:

2 $$\frac{{\sigma }_{{{{{{\rm{SC}}}}}}}(T)}{{\gamma }_{\mu }}=0.06091\frac{{\Phi }_{0}}{{\lambda }^{2}(T)},$$

where *γ*_*μ*_ is the gyromagnetic ratio of the muon and Φ_0_ is the magnetic-flux quantum. Since the applied field is a factor of ~30 times smaller than the second critical magnetic fields in RbV_3_Sb_5_, this equation is valid to estimate the *λ*.

### Pressure cell

Pressures up to 1.9 GPa were generated in a double wall piston-cylinder type cell made of CuBe/MP35N, specially designed to perform *μ*SR experiments under pressure^59^. A fully assembled typical double-wall pressure cell is presented and discussed in Supplementary Fig. 1 and Supplementary Note 1. The body of the pressure cell consists of two parts: the inner and the outer cylinders which are shrink fitted into each other. Outer body of the cell is made out of MP35N alloy. Inner body of the cell is made out of CuBe alloy. Other components of the cell are: pistons, mushroom, seals, locking nuts, and spacers. The mushroom pieces and sealing rings were made out of non hardened Copper Beryllium. With both pistons completely inserted, the maximum sample height is 12 mm. As a pressure transmitting medium Daphne oil was used. The pressure was measured by tracking the superconducting transition of a very small indium plate by AC susceptibility. The filling factor of the pressure cell was maximized. The fraction of the muons stopping in the sample was approximately 40%.

### Crystal structure of RbV_3_Sb_5_

Additional characterization information is provided here on the kagome superconductor RbV_3_Sb_5_ which crystallizes in the novel *A*V_3_Sb_5_-type structure (space group *P*6/*mmm*, where *A* = K, Rb, Cs). The crystallographic structure of prototype compound RbV_3_Sb_5_ shown in panel (a) of Supplementary Fig. 2 (see Supplementary Note 2) illustrates how the V atoms form a kagome lattice (medium beige circles) intertwined with a hexagonal lattice of Sb atoms (small red circles). The Rb atoms (large purple circles) occupy the interstitial sites between the two parallel kagome planes. In panel (b) the vanadium kagome net has been emphasized, with the interpenetrating antimony lattice included to highlight the unit cell (see dashed lines). Supplementary Fig. 2c shows an optical microscope image of several single crystals of RbV_3_Sb_5_ on millimeter paper. The Laue X-ray diffraction image (see Supplementary Fig. 2d) demonstrates the single crystallinity of the samples used for *μ*SR experiments.

### Analysis of high-field TF-*μ*SR data

Figure 1g shows the probability field distribution, measured at 3 K for the single-crystal samples of RbV_3_Sb_5_ in the *c*-axis magnetic field of 8 T. In the whole investigated temperature range, two-component signals were observed: a signal with fast relaxation (low frequency) and another one with a slow relaxation (high frequency). The narrow signal arises mostly from the muons stopping in the silver sample holder and its position is a precise measure of the value of the applied magnetic field. The width and the position of the narrow signal is assumed to be temperature independent and thus they were kept constant in the analysis. The relative fraction of the muons stopping in the sample was fixed to the value obtained at the base-*T* and kept temperature independent. The signal with the fast relaxation, which is shifted towards the lower field from the applied one, arises from the muons stopping in the sample and it takes a major fraction (80%) of the *μ*SR signal. This points to the fact that the sample response arises from the bulk of the sample. We note that in high magnetic fields we cannot systematically discriminate between the nuclear and the electronic contribution to the relaxation rate and thus we show the total high-field muon spin relaxation rate *σ*_HTF_ in Fig. 1i.

### Analysis of ZF-*μ*SR data under pressure

As an example, in Supplementary Fig. 6 (see Supplementary Note 6) is displaying the zero-field *μ*SR spectra, recorded at *p* = 1.07 GPa for various temperatures. The experimental data were analyzed by separating the *μ*SR signal on the sample (s) and the pressure cell (pc) contributions^51^:

3 $${A}_{0}P(t)={A}_{{{{{{{{\rm{s}}}}}}}}}{P}_{{{{{{{{\rm{s}}}}}}}}}(t)+{A}_{{{{{{{{\rm{pc}}}}}}}}}{P}_{{{{{{{{\rm{pc}}}}}}}}}(t).$$

Here *A*_0_ is the initial asymmetry of the muon-spin ensemble, and *A*_s_ (*A*_pc_) and *P*_s_(*t*) [*P*_pc_(*t*)] are the asymmetry and the time evolution of the muon-spin polarization for muons stopped inside the sample (pressure cell), respectively. The response of the pressure cell [*P*_pc_(*t*)] was studied in separate set of experiments.

The sample contribution includes both, the nuclear moment and an additional exponential relaxation Γ caused by appearance of spontaneous magnetic fields:

4 $${P}_{{{{{{{{\rm{s}}}}}}}}}^{{{{{{{{\rm{ZF}}}}}}}}}(t)={P}_{{{{{{\rm{ZF}}}}}}}^{{{{{{\rm{GKT}}}}}}}(t){e}^{-\Gamma t}.$$

Here ${P}_{{{{{{\rm{ZF}}}}}}}^{{{{{{\rm{GKT}}}}}}}(t)$ is the GKT relaxation function (see Eq. (1)) describing the magnetic field distribution created by the nuclear magnetic moments^44^. Fits of Eq. (3) to the ZF-*μ*SR pressure data were performed globally. The ZF-*μ*SR time-spectra taken at each particular pressure (*p* = 0.16, 0.59, 1.07, 1.53, and 1.89 GPa) were fitted simultaneously with *A*_s_, *A*_pc_, and *σ*_GKT_ as common parameters, and *λ* an individual parameter for each particular data set. The fits were limited to *T* ≃ 150 K, i.e., up to the temperature where the nuclear contribution of RbV_3_Sb_5_ remains constant (*σ*_GKT_ ≃ const, see Fig. 1e).

### Macroscopic superconducting properties under pressure

The temperature dependence of the AC-susceptibility *χ*_AC_ for various pressures for the polycrystalline samples of RbV_3_Sb_5_ and KV_3_Sb_5_ are shown in Supplementary Fig. 8a, b (see Supplementary Note 8). We kept the position of the AC coil, mounted on the pressure cell, the same for the measurements at various applied pressures in order to be able to directly compare the superconducting responses at various applied pressures in both RbV_3_Sb_5_ and KV_3_Sb_5_. Moreover, we used the same amount of RbV_3_Sb_5_ and KV_3_Sb_5_ samples. The data for RbV_3_Sb_5_ at the pressure 1.45 GPa, where *T*_c_ reaches the maximum shows sharp superconducting transition with saturated full superconducting screening. We used the maximum value of the diamagnetic susceptibility at 1.45 GPa and normalize the rest of the data by that. Our results indicate a strong diamagnetic response and sharp superconducting transitions in both samples. This points to the high quality of the samples and providing evidence for bulk superconductivity in these polycrystalline samples.

### Analysis of *λ*(*T*)

*λ*(*T*) was calculated within the local (London) approximation (*λ* ≫ *ξ*) by the following expression^60^ (see Supplementary Notes 9 and 10 and Supplementary Fig. 9):

5 $$\frac{{\lambda }^{-2}(T,{\Delta }_{0,i})}{{\lambda }^{-2}(0,{\Delta }_{0,i})}=1+\frac{1}{\pi }\int\nolimits_{0}^{2\pi }\int\nolimits_{{\Delta }_{i}(T,\varphi )}^{\infty }\left(\frac{\partial f}{\partial E}\right)\frac{EdEd\varphi }{\sqrt{{E}^{2}-{\Delta }_{i}{(T,\varphi )}^{2}}},$$

where $f={[1+\exp (E/{k}_{{{{{{{{\rm{B}}}}}}}}}T)]}^{-1}$ is the Fermi function, *φ* is the angle along the Fermi surface, and Δ_*i*_(*T*, *φ*) = Δ_0,*i*_Γ(*T*/*T*_c_)*g*(*φ*) (Δ_0,*i*_ is the maximum gap value at *T* = 0). The temperature dependence of the gap is approximated by the expression $\Gamma (T/{T}_{{{{{{{{\rm{c}}}}}}}}})=\tanh \{1.82{[1.018 ({T}_{{{{{{{{\rm{c}}}}}}}}}/T-1)]}^{0.51}\}$,^61^ while *g*(*φ*) describes the angular dependence of the gap and it is replaced by 1 for both an *s*-wave and an *s*+*s*-wave gap, $|\cos (2\varphi )|$ for a *d*-wave gap, and $|\cos (6\varphi )|$ for a *f*-wave gap.

For RbV_3_Sb_5_ and KV_3_Sb_5_, the *λ*^−2^(*T*) data above ${p}_{\max -{{{{{{{\rm{Tc}}}}}}}}}$ are analyzed using two *s*-wave gaps. At pressure below ${p}_{\max -{{{{{{{\rm{Tc}}}}}}}}}$, the combination of dominant nodal $|\cos (6\varphi )|$-gap and one *s*-wave gap is used.

### Analysis of the temperature dependence of the penetration depth for the single crystals RbV_3_Sb_5_ and KV_3_Sb_5_ at ambient pressure

${\lambda }_{{{{{{\rm{eff}}}}}}}^{-2}(T)$ at ambient pressure were analyzed within the framework of quasi-classical Eilenberger weak-coupling formalism, where the temperature dependence of the gaps was obtained by solving self-consistent coupled gap equations rather than using the phenomenological *α*-model, where the latter considers a similar BCS-type temperature dependence for both gaps (see Supplementary Note 10 and Supplementary Fig. 10). This method is described in details elsewhere^62–64^, including in our recent paper on KV_3_Sb_5_^25^. The temperature dependence of ${\lambda }_{ab}^{-2}$ down to 18 mK in the applied field of 5 mT is shown in Supplementary Fig. 9 for RbV_3_Sb_5_ along with the KV_3_Sb_5_ data. A well-pronounced two-step behavior is observed in RbV_3_Sb_5_, similar to KV_3_Sb_5_^25^. Our numerical analysis allows to determine the interband coupling and the superconducting gap values. The analysis reveals that the two step transition in *σ*_sc_(*T*) at 5 mT requires the interband coupling constant to be small, 0.001. The small values of interband coupling constants imply that the band(s), where the large and the small superconducting energy gaps are open, become only weakly coupled. One important point is that if we assume the maximum gap-to-*T*_c_ ratio to be 3.75 (BCS value), then one can not reproduce the sharp step-like feature in *σ*_sc_(*T*). The data are well explained by a large value of 2Δ/*k*_B_*T*_c_ ≃ 7. Our observation of two step behavior of penetration depth in the system KV_3_Sb_5_ with single *T*_c_ is consistent with two gap superconductivity with very weak interband coupling and large value of 2Δ/*k*_B_*T*_c_ ≃ 7. The interband coupling is extremely small which is sufficient to have same values of *T*_c_ for different bands but still shows the two step temperature behavior of the penetration depth^65^. The ${\lambda }_{ab}^{-2}(T)$ for both (Rb,K)V_3_Sb_5_ are well described by one constant gap and one dominant angle-dependent $|\cos (6\varphi )|$-gap, indicating the presence of gap nodes. Upon increasing pressure two step behavior gets smoothed out, but angle-dependent gap becomes more dominant and persists all the way up to ${p}_{\max -{{{{{{{\rm{Tc}}}}}}}}}\simeq {p}_{{{{{{{{\rm{cr,co}}}}}}}}}\simeq$ 1.5 and 0.5 GPa for RbV_3_Sb_5_ and KV_3_Sb_5_, respectively. At pressures above ${p}_{\max -{{{{{{{\rm{Tc}}}}}}}}}$, the *λ*^−2^(*T*) is described by constant gaps.

### Analysis of TF-*μ*SR data under pressure

The TF *μ*SR data were analyzed by using the following functional form^60^:

6 $$P(t)=	 {A}_{s}\exp \left[-\frac{({\sigma }_{{{{{{{{\rm{sc}}}}}}}}}^{2}+{\sigma }_{{{{{{{{\rm{nm}}}}}}}}}^{2}){t}^{2}}{2}\right]\cos ({\gamma }_{\mu }{B}_{{{{{{{{\rm{int}}}}}}}},{{{{{{{\rm{s}}}}}}}}}t+\varphi )\\ 	+{A}_{{{{{{{{\rm{pc}}}}}}}}}\exp \left[-\frac{{\sigma }_{{{{{{{{\rm{pc}}}}}}}}}^{2}{t}^{2}}{2}\right]\cos ({\gamma }_{\mu }{B}_{{{{{{{{\rm{int}}}}}}}},{{{{{{{\rm{pc}}}}}}}}}t+\varphi ),$$

here *A*_s_ and *A*_pc_ denote the initial asymmetries of the sample and the pressure cell, respectively. *φ* is the initial phase of the muon-spin ensemble and *B*_int_ represents the internal magnetic field at the muon site. The relaxation rates *σ*_sc_ and *σ*_nm_ characterize the damping due to the formation of the FLL in the superconducting state and of the nuclear magnetic dipolar contribution, respectively. In the analysis *σ*_nm_ was assumed to be constant over the entire temperature range and was fixed to the value obtained above *T*_c_ where only nuclear magnetic moments contribute to the muon depolarization rate *σ*. The Gaussian relaxation rate, *σ*_pc_, reflects the depolarization due to the nuclear moments of the pressure cell. The width of the pressure cell signal increases below *T*_*c*_ (see Supplementary Note 12 and Supplementary Fig. 10). This is due to the influence of the diamagnetic moment of the superconducting sample on the pressure cell, leading to the temperature dependent *σ*_pc_ below *T*_c_. In order to consider this influence we assume the linear coupling between *σ*_pc_ and the field shift of the internal magnetic field in the superconducting state:

7 $${\sigma }_{{{{{{{{\rm{pc}}}}}}}}}(T)={\sigma }_{{{{{{{{\rm{pc}}}}}}}}}(T \, > \, {T}_{{{{{{{{\rm{c}}}}}}}}})+C({\mu }_{{{{{{{{\rm{0}}}}}}}}}{H}_{{{{{{{{\rm{int}}}}}}}},{{{{{{{\rm{NS}}}}}}}}}-{\mu }_{{{{{{{{\rm{0}}}}}}}}}{H}_{{{{{{{{\rm{int}}}}}}}},{{{{{{{\rm{SC}}}}}}}}}(T)),$$

where *σ*_pc_(*T* > *T*_c_) = 0.25 *μ*s^−1^ is the temperature-independent Gaussian relaxation rate. *μ*_0_*H*_int,NS_ and *μ*_0_*H*_int,SC_ are the internal magnetic fields measured in the normal and in the superconducting state, respectively. As indicated by the solid lines in Supplementary Fig. 10a–d, the *μ*SR data are well described by Eqs. (6) and (7).

## Supplementary information

Supplementary Information

## Data Availability

All relevant data are available from the authors. Alternatively, the data can be accessed through the database at the following link http://musruser.psi.ch/cgi-bin/SearchDB.cgi.
