# Supplementary material for: Tunable unconventional kagome superconductivity in charge ordered RbV3Sb5 and KV3Sb5
Source: Nat Commun. 2023 Jan 11;14:153. doi: 10.1038/s41467-022-35718-z (PMC9834244; doi:10.1038/s41467-022-35718-z)
Supplement: Supplementary file 1 — Supplementary Information [file 41467_2022_35718_MOESM1_ESM.pdf]

preprint(December 13, 2022)

Supplementary Information: Tunable unconventional kagome superconductivity in charge ordered RbV_3Sb_5 and KV_3Sb_5

Z. Guguchia,[1,*] C. Mielke III,[1] D. Das,[1] R. Gupta,[1] J.-X. Yin,[2] H. Liu,[3,4] Q. Yin,[5] M.H. Christensen,[6] Z. Tu,[5] C. Gong,[5] N. Shumiya,[2] Md Shafayat Hossain,[2] Ts. Gamsakhurdashvili,[1] M. Elender,[1] Pengcheng Dai,[7] A. Amato,[1] Y. Shi,[3,4] H.C. Lei,[5] R.M. Fernandes,[8] M.Z. Hasan,[2,9,10,11] H. Luetkens,[1,†] and R. Khasanov[1,‡]

[1]*Laboratory for Muon Spin Spectroscopy, Paul Scherrer Institute, CH-5232 Villigen PSI, Switzerland*

[2]*Laboratory for Topological Quantum Matter and Advanced Spectroscopy (B7), Department of Physics, Princeton University, Princeton, New Jersey 08544, USA*

[3]*Beijing National Laboratory for Condensed Matter Physics and Institute of Physics, Chinese Academy of Sciences, Beijing 100190, China.*

[4]*University of Chinese Academy of Sciences, Beijing 100049, China.*

[5]*Department of Physics and Beijing Key Laboratory of Opto-electronic Functional Materials and Micro-nano Devices, Renmin University of China, Beijing 100872, China*

[6]*Niels Bohr Institute, University of Copenhagen, 2100 Copenhagen, Denmark*

[7]*Department of Physics and Astronomy, Rice Center for Quantum Materials, Rice University, Houston, TX, USA*

[8]*School of Physics and Astronomy, University of Minnesota, Minneapolis, MN 55455, USA*

[9]*Princeton Institute for the Science and Technology of Materials, Princeton University, Princeton, New Jersey 08540, USA*

[10]*Materials Sciences Division, Lawrence Berkeley National Laboratory, Berkeley, California 94720, USA*

[11]*Quantum Science Center, Oak Ridge, Tennessee 37831, USA*

I. SUPPLEMENTARY NOTE 1: PRESSURE CELL

Pressures up to 1.9 GPa were generated in a double wall piston-cylinder type cell made of CuBe/MP35N, specially designed to perform μSR experiments under pressure [1]. A fully assembled typical double-wall pressure cell is presented in Supplementary Fig. 1. The body of the pressure cell consists of two parts: the inner and the outer cylinders which are shrink fitted into each other. Outer body of the cell is made out of MP35N alloy. Inner body of the cell is made out of CuBe alloy. Other components of the cell are: pistons, mushroom, seals, locking nuts, and spacers. The mushroom pieces and sealing rings were made out of non hardened Copper Beryllium. With both pistons completely inserted, the maximum sample height is 12 mm. As a pressure transmitting medium Daphne oil was used. The pressure was measured by tracking the superconducting transition of a very small indium plate by AC susceptibility. The filling factor of the pressure cell was maximized. The fraction of the muons stopping in the sample was approximately 40 %.

II. SUPPLEMENTARY NOTE 2: CRYSTAL STRUCTURE OF RBV_3SB_5

Additional characterization information is provided here on the kagome superconductor RbV_3Sb_5 which crystallizes in the novel AV_3Sb_5-type structure (space group $P6/mmm$, where A = K, Rb, Cs). The crystallographic structure of prototype compound RbV_3Sb_5 shown in panel (a) of Supplementary Figure 2 illustrates how the V atoms form a kagome lattice (medium beige circles) intertwined with a hexagonal lattice of Sb atoms (small red circles). The Rb atoms (large purple circles) occupy the interstitial sites between the two parallel kagome planes. In panel (b) the vanadium kagome net has been emphasized, with the interpenetrating antimony lattice included to highlight the unit cell (see dashed lines). Supplementary Figures 2c shows an optical microscope image of several single crystals of RbV_3Sb_5 on millimeter paper. The Laue X-ray diffraction image (see the Supplementary Figure 2d) demonstrates the single crystallinity of the samples used for μSR experiments.

III. SUPPLEMENTARY NOTE 3: MAGNETIZATION MEASUREMENTS OF RbV_3Sb_5

The magnetization measurements show the abrupt drop in macroscopic magnetization across $T_1^* = T_{\mathrm{CDW},1} \simeq 105$ K for the field applied along the c-axis, as shown in Supplementary Figure 3. Interestingly, a shallow minimum around $T_2^* = T_{\mathrm{CDW},2} \simeq 50$ K is also seen in magnetization, followed by sizeable increase at lower temperatures.

IV. SUPPLEMENTARY NOTE 4: STATIC NATURE OF INTERNAL FIELDS IN THE SUPERCONDUCTING STATE OF RbV_3Sb_5

Supplementary Figure 4 shows the ZF-μSR time spectra for RbV_3Sb_5, obtained at $T =$ 0.28 K. Moreover, the μSR spectra obtained in longitudinally-applied field (LF) of 50 G both at 0.28 K and 5 K are also shown. Because the zero-field relaxation is clearly decoupled by the application of a small external magnetic field applied in a direction longitudinal to the muon spin polarization, the relaxation is therefore due to spontaneous fields that are static at the microsecond timescale.

V. SUPPLEMENTARY NOTE 5: KNIGHT SHIFT OF RbV_3Sb_5

Supplementary Figure 5 shows the the temperature dependence of the Knight shift, measured at various applied magnetic fields. Knight shift is defined as $K_{\mathrm{exp}} = (B_{\mathrm{int}} - B_{\mathrm{ext}})/B_{\mathrm{ext}}$, where B_{int} and B_{ext} are the internal and externally applied magnetic fields, respectively. K_{exp} shows a sharp changes across T_1^* and T_2^*, which indicates the change of local magnetic susceptibility with two characteristic temperatures.

VI. SUPPLEMENTARY NOTE 6: ANALYSIS OF ZF-μSR DATA UNDER PRESSURE

As an example, in the Supplementary Figure 6 is displaying the zero-field μSR spectra, recorded at $p = 1.07$ GPa for various temperatures. The experimental data were analyzed

by separating the μSR signal on the sample (s) and the pressure cell (pc) contributions [2, 3]:

$$A_0P(t) = A_{\rm s}P_{\rm s}(t) + A_{\rm pc}P_{\rm pc}(t). \tag{1}$$

Here A_0 is the initial asymmetry of the muon-spin ensemble, and $A_{\rm s}$ ($A_{\rm pc}$) and $P_{\rm s}(t)$ [$P_{\rm pc}(t)$] are the asymmetry and the time evolution of the muon-spin polarization for muons stopped inside the sample (pressure cell), respectively. The response of the pressure cell [$P_{\rm pc}(t)$] was studied in separate set of experiments.

The sample contribution includes both, the nuclear moment and an additional exponential relaxation Γ caused by appearance of spontaneous magnetic fields:

$$P_{\rm s}^{\rm ZF}(t) = P_{ZF}^{GKT}(t)e^{-\Gamma t}. \tag{2}$$

Here $P_{ZF}^{GKT}(t)$ is the Gaussian Kubo-Toyabe (GKT) relaxation function (see Eq. 1) describing the magnetic field distribution created by the nuclear magnetic moments [4]. Fits of Eq. 1 to the ZF-μSR pressure data were performed globally. The ZF-μSR time-spectra taken at each particular pressure ($p = 0.16$, 0.59, 1.07, 1.53, and 1.89 GPa) were fitted simultaneously with $A_{\rm s}$, $A_{\rm pc}$, and $\sigma_{\rm GKT}$ as common parameters, and λ an individual parameter for each particular data set. The fits were limited to $T \simeq 150$ K, *i.e.* up to the temperature where the nuclear contribution of RbV_3Sb_5 remains constant ($\sigma_{\rm GKT} \simeq const$, see Fig. 1e).

VII. SUPPLEMENTARY NOTE 7: TIME-REVERSAL SYMMETRY-BREAKING CHARGE ORDERS UNDER PRESSURE

Here we show (see Supplementary Figure 7a-f) the evolution of the two time-reversal symmetry-breaking transition temperatures T_1^* and T_2^* with the application of hydrostatic pressure. Two step time-reversal symmetry-breaking transition is clearly observed under the pressures of $p = 0.16$ GPa and 0.59 GPa. At 1 GPa, these two transitions become indistinguishable and above 1 GPa we see only transition at T_2^*, which decreases upon further increasing the pressure. Supplementary Figure 7f shows the pressure evolution of T_1^* and T_2^*, extracted from μSR results, and of previously reported charge order temperature $T_{\rm co,1}$ [5]. The value of $T_{\rm co,2}$ [6] at ambient pressure is also shown. This phase diagram shows that two time-reversal symmetry-breaking state turn into single time-reversal symmetry-breaking state at ~ 1.5 GPa, above which T_2^* shows faster suppression and follows the phase line of the

charge order. Thus, this phase diagram suggests three distinct pressure regions: (a) Pressure range between 0 GPa and 1.5 GPa in which two charge order transitions are observed. (b) Pressure range between 1.5 GPa and 2.2 GPa in which only one TRS breaking charge order transition is observed. (c) Pressures above 2.2 GPa [5] where charge order is fully suppressed. Interestingly, $p_{\mathrm{max-Tc}} = 1.5$ GPa is the pressure at which T_{c} reaches its maximum value. Also, the fact that the pressure dependence of the time-reversal symmetry-breaking temperature matches well with the pressure evolution of the charge order temperature confirms the charge order being responsible for the time-reversal symmetry-breaking in RbV_3Sb_5.

VIII. SUPPLEMENTARY NOTE 8: MACROSCOPIC SUPERCONDUCTING PROPERTIES UNDER PRESSURE

The temperature dependence of the AC-susceptibility χ_{AC} for various pressures for the polycrystalline samples of RbV_3Sb_5 and KV_3Sb_5 are shown in Supplementary Figures 8a and b. We kept the position of the AC coil, mounted on the pressure cell, the same for the measurements at various applied pressures in order to be able to directly compare the superconducting responses at various applied pressures in both RbV_3Sb_5 and KV_3Sb_5. Moreover, we used the same amount of RbV_3Sb_5 and KV_3Sb_5 samples. The data for RbV_3Sb_5 at the pressure 1.45 GPa, where T_{c} reaches the maximum shows sharp superconducting transition with saturated full superconducting screening. We used the maximum value of the diamagnetic susceptibility at 1.45 GPa and normalise the rest of the data by that. Our results indicate a strong diamagnetic response and sharp superconducting transitions in both samples. This points to the high quality of the samples and providing evidence for bulk superconductivity in these polycrystalline samples.

IX. SUPPLEMENTARY NOTE 9: ANALYSIS OF $\lambda(T)$

$\lambda(T)$ was calculated within the local (London) approximation ($\lambda \gg \xi$) by the following expression [8–10]:

$$\frac{\lambda^{-2}(T,\Delta_{0,i})}{\lambda^{-2}(0,\Delta_{0,i})} = 1 + \frac{1}{\pi}\int_0^{2\pi}\int_{\Delta_i(T,\varphi)}^{\infty}\left(\frac{\partial f}{\partial E}\right)\frac{E dE d\varphi}{\sqrt{E^2-\Delta_i(T,\varphi)^2}}, \tag{3}$$

where $f = [1 + \exp(E/k_{\rm B}T)]^{-1}$ is the Fermi function, φ is the angle along the Fermi surface, and $\Delta_i(T,\varphi) = \Delta_{0,i}\Gamma(T/T_{\rm c})g(\varphi)$ ($\Delta_{0,i}$ is the maximum gap value at $T = 0$). The temperature dependence of the gap is approximated by the expression $\Gamma(T/T_{\rm c}) = \tanh\{1.82[1.018(T_{\rm c}/T - 1)]^{0.51}\}$,[11] while $g(\varphi)$ describes the angular dependence of the gap and it is replaced by 1 for both an s-wave and an s+s-wave gap, $|\cos(2\varphi)|$ for a d-wave gap, and $|\cos(6\varphi)|$ for a f-wave gap.

For RbV_3Sb_5 and KV_3Sb_5, the $\lambda^{-2}(T)$ data above $p_{\rm max-Tc}$ are analysed using two s-wave gaps. At pressure below $p_{\rm max-Tc}$, the combination of dominant nodal $|\cos(6\varphi)|$-gap and one s-wave gap is used.

X. SUPPLEMENTARY NOTE 10: ANALYSIS OF THE TEMPERATURE DEPENDENCE OF THE PENETRATION DEPTH FOR THE SINGLE CRYSTALS RBV_3SB_5 AND KV_3SB_5 AT AMBIENT PRESSURE

$\lambda_{eff}^{-2}(T)$ at ambient pressure were analyzed within the framework of quasi-classical Eilenberger weak-coupling formalism, where the temperature dependence of the gaps was obtained by solving self-consistent coupled gap equations rather than using the phenomenological α-model, where the latter considers a similar BCS-type temperature dependence for both gaps. This method is described in details elsewhere [12–15], including in our recent paper on KV_3Sb_5 [16]. The temperature dependence of λ_{ab}^{-2} down to 18 mK in the applied field of 5 mT is shown in Supplementary Figure 9 for RbV_3Sb_5 along with the KV_3Sb_5 data. A well pronounced two step behaviour is observed in RbV_3Sb_5, similar to KV_3Sb_5 [16]. Our numerical analysis allows to determine the interband coupling and the superconducting gap values. The analysis reveals that the two step transition in $\sigma_{\rm sc}(T)$ at 5 mT requires the interband coupling constant to be small, 0.001. The small values of interband coupling constants imply that the band(s), where the large and the small superconducting energy gaps are open, become only weakly coupled. One important point is that if we assume the maximum gap-to-$T_{\rm c}$ ratio to be 3.75 (BCS value), then one can not reproduce the sharp step-like feature in $\sigma_{\rm sc}(T)$. The data are well explained by a large value of $2\Delta/k_{\rm B}T_{\rm c} \simeq 7$. Our observation of two step behaviour of penetration depth

in the system KV_3Sb_5 with single T_c is consistent with two gap superconductivity with very weak interband coupling and large value of $2\Delta/k_BT_c \simeq 7$. The interband coupling is extremely small which is sufficient to have same values of T_c for different bands but still shows the two step temperature behaviour of the penetration depth [17]. The $\lambda_{ab}^{-2}(T)$ for both (Rb,K)V_3Sb_5 are well described by one constant gap and one dominant angle-dependent $|\cos(6\varphi)|$-gap, indicating the presence of gap nodes. Upon increasing pressure two step behaviour gets smoothed out, but angle-dependent gap becomes more dominant and persists all the way up to $p_{\rm max-Tc} \simeq p_{\rm cr,co} \simeq 1.5$ GPa and 0.5 GPa for RbV_3Sb_5 and KV_3Sb_5, respectively. At pressures above $p_{\rm max-Tc}$, the $\lambda^{-2}(T)$ is described by constant gaps.

XI. SUPPLEMENTARY NOTE 11: TF-μSR SPECTRA FOR RBV_3SB_5 AND KV_3SB_5

Supplementary Figures. 10a and b show the TF-μSR spectra, measured near ambient pressure above and below the superconducting transition temperature T_c for RbV_3Sb_5 and KV_3Sb_5, respectively. Supplementary Figures. 10c and d show the TF-μSR spectra, measured above and below the superconducting transition temperature T_c for RbV_3Sb_5 at $p =$ 1.85 GPa and for KV_3Sb_5 at $p = 1.1$ GPa, respectively. In order to obtain well ordered vortex lattice, the measurements were done after field cooling the sample from above T_c. Above T_c, the oscillations show a damping essentially due to the random local fields from the nuclear magnetic moments. Below T_c the damping rate increases with decreasing temperature due to the presence of a nonuniform local magnetic field distribution as a result of the formation of a flux-line lattice in the superconducting state. Figures 10c and d show that damping in the superconducting state significantly increases upon application of hydrostatic pressure.

XII. SUPPLEMENTARY NOTE 12: ANALYSIS OF TF-μSR DATA UNDER PRESSURE

The TF μSR data were analyzed by using the following functional form:[8]

$$\begin{aligned} P(t) = A_s \exp\Big[-\frac{(\sigma_{\rm sc}^2+\sigma_{\rm nm}^2)t^2}{2}\Big]\cos(\gamma_\mu B_{\rm int,s}t+\varphi) \\ +A_{\rm pc}\exp\Big[-\frac{\sigma_{\rm pc}^2 t^2}{2}\Big]\cos(\gamma_\mu B_{\rm int,pc}t+\varphi), \end{aligned} \tag{4}$$

Here $A_{\rm s}$ and $A_{\rm pc}$ denote the initial assymmetries of the sample and the pressure cell, respectively. φ is the initial phase of the muon-spin ensemble and $B_{\rm int}$ represents the internal magnetic field at the muon site. The relaxation rates $\sigma_{\rm sc}$ and $\sigma_{\rm nm}$ characterize the damping due to the formation of the FLL in the superconducting state and of the nuclear magnetic dipolar contribution, respectively. In the analysis $\sigma_{\rm nm}$ was assumed to be constant over the entire temperature range and was fixed to the value obtained above $T_{\rm c}$ where only nuclear magnetic moments contribute to the muon depolarization rate σ. The Gaussian relaxation rate, $\sigma_{\rm pc}$, reflects the depolarization due to the nuclear moments of the pressure cell. The width of the pressure cell signal increases below T_c. This is due to the influence of the diamagnetic moment of the superconducting sample on the pressure cell, leading to the temperature dependent $\sigma_{\rm pc}$ below T_c. In order to consider this influence we assume the linear coupling between $\sigma_{\rm pc}$ and the field shift of the internal magnetic field in the superconducting state:

$$\sigma_{\rm pc}(T) = \sigma_{\rm pc}(T{>}T_{\rm c}) + C(\mu_0 H_{\rm int,NS} - \mu_0 H_{\rm int,SC}(T)), \tag{5}$$

where $\sigma_{\rm pc}(T > T_{\rm c}) = 0.25\ \mu s^{-1}$ is the temperature independent Gaussian relaxation rate. $\mu_0 H_{\rm int,NS}$ and $\mu_0 H_{\rm int,SC}$ are the internal magnetic fields measured in the normal and in the superconducting state, respectively. As indicated by the solid lines in Supplementary Figs. 10a-d the μSR data are well described by Eqs. (4-5).

XIII. SUPPLEMENTARY NOTE 13: COMPARISON BETWEEN NODAL AND NODELESS SUPERCONDUCTING GAP MODELS AT VARIOUS PRESSURES

For clarity purposes, here we show the temperature dependences of the superconducting muon spin relaxation rate $\sigma_{\rm sc}$ individually for various pressures (see the Supplementary

Figures 11-12). For each selected pressure, we show the fit results using a model with nodal and nodeless superconductivity.

For the RbV_3Sb_5 system, the pressure at which the superconducting transition temperature reaches its maximum value is $p_{\mathrm{max-Tc}} \simeq 1.58$ GPa, which is lower than the critical pressure $p_{\mathrm{cr}} \simeq 2.2$ GPa at which the charge order is fully supressed. This is different from KV_3Sb_5 in which $p_{\mathrm{max-Tc}} \simeq p_{\mathrm{cr}}$. Our quantitative analysis shows that at pressures below $p_{\mathrm{max-Tc}} \simeq 1.58$ GPa (see the Supplementary Figures 11a-b), the temperature dependence of the penetration depth in RbV_3Sb_5 is very well described by the model with a nodal gap. At the applied pressures of 1.85 GPa and 2.25 GPa, the analysis clearly show that at least ten points below 2 K do not follow the linear temperature dependence (see the Supplementary Figures 11c). The temperature dependence is much better described by a model with a nodeless gap. This conclusion is supported by a ${\chi_r}^2$-comparison, revealing a value of ${\chi_r}^2$ for the nodal gap model that is higher by factor of ∼3.9 than the one for the nodeless gap model. There is one point at the base-T for 1.85 GPa that deviates from the saturating behaviour. This is similar to what we see in the Supplementary Figures 12a-f) for the KV_3Sb_5 system around 0.72 GPa and 1.1 GPa. This feature is therefore consistent with the fact that the pressure of 1.85 GPa for RbV_3Sb_5 is not sufficiently large to fully suppress the charge order in RbV_3Sb_5. But at the highest applied pressure of 2.25 GPa where charge order is fully suppressed, the data display clear saturation (see the Supplementary Figure 11d). So, the results indicate that in RbV_3Sb_5 a clear deviation from nodal behaviour starts at pressures above $p_{\mathrm{max-Tc}} \simeq 1.58$ GPa and becomes unambiguously nodeless after fully suppressing the charge order.

For KV_3Sb_5 (see the Supplementary Figures 12a-f), the temperature dependence of the penetration depth at $p = 0.36$ GPa is very well captured by the model with a nodal gap. The data at 0.42 GPa which is close to the critical pressure for the suppression of charge order $p_{\mathrm{cr}} \simeq 0.5$ GPa, is also better described by the model with a nodal gap (points below 1.2 K follow the linear temperature dependence). This is confirmed quantitatively from the fact that the reduced ${\chi_r}^2$ for the nodal gap model is lower by factor of ∼1.4 than the one for nodeless gap. However, at $p = 0.64$ GPa, which is above $p_{\mathrm{cr}} \simeq 0.5$ GPa, the data points below 1.2 K do not follow the linear temperature dependence and are better

described by a model with a nodeless gap. In this case, ${\chi_r}^2$ for the nodal gap model is higher by factor of ∼1.2 than the one for nodeless gap. Upon further increasing pressure, the deviation from the nodal behaviour becomes more pronounced. Namely, for $p = 0.72$ GPa and 1.1 GPa, ${\chi_r}^2$ for the nodal gap model is higher by factor of ∼1.8 than the one for the nodeless gap. At 1.58 GPa, ${\chi_r}^2$ for the nodal gap model is higher by factor of ∼2.65 than the one for nodeless gap, and the data display clear saturation. These results show that the superconducting state of KV_3Sb_5 starts deviating from the nodal behaviour above $p_{\rm cr} \simeq 0.5$ GPa and becomes unambiguously nodeless well into the suppressed charge ordered state. We acknowledge that in the intermediate regime between unambiguously nodal ($p = 0.36$ GPa) and unambiguously nodeless ($p = 1.5$ GPa), and particularly near the latter, the lowest temperature data point seems to deviate from the best fitting curve. The nature of this deviation is unclear - larger statistical scattering at low temperatures or intrinsic crossover behavior from nodal to nodeless. Nevertheless, the data at the two extreme points show clearly that a transition from nodal to nodeless must necessarily take place for an intermediate pressure value.

To summarise, our results demonstrate the nodal superconducting pairing in both KV_3Sb_5 and RbV_3Sb_5 below $p_{\rm max-Tc}$. While for KV_3Sb_5 there is unambiguous evidence for nodeless superconductivity once charge order is completely suppressed, for RbV_3Sb_5 there is very strong indication that a change to nodeless behavior starts at around $p_{\rm max-Tc}$. This is quantitatively shown in in the Supplementary Figure 13, where we plot the pressure dependence of the ratio ${\chi_{\rm nodal}}^2/{\chi_{\rm nodeless}}^2$, crossing the ${\chi_{\rm nodal}}^2/{\chi_{\rm nodeless}}^2 = 1$ line at $p_{\rm max-Tc}$.

XIV. SUPPLEMENTARY NOTE 14: THEORETICAL MODEL FOR THE NODAL-TO-NODELESS TRANSITION

Here we present more details about our theoretical model for the nodal-to-nodeless transition assuming that the "pure" superconducting state is the time-reversal symmetry-breaking chiral $d_{x^2-y^2} + id_{xy}$ state. The kagome lattice has point group D_{6h} and the d-wave order parameter has two degenerate components corresponding to the $\Delta_{d_{x^2-y^2}} = \Delta_1$ and the

Solution	ϕ_*	θ_*	$\Delta_{0,*}$	$\mathcal{F}_*$
Disordered	–	–	0	0
Nodal (1)	-	$\frac{\pi}{2}$	$\sqrt{\frac{-\alpha-\lambda\Delta_{\rm CO}^2}{2\beta_1}}$	$-\frac{(\alpha-\lambda\Delta_{\rm CO}^2)^2}{4\beta_1}$
Nodeless (2)	$\frac{\pi}{2}$	$\frac{1}{2}\arctan\left[\frac{\sqrt{4\beta_2^2\Delta_0^2-\lambda^2\Delta_{\rm CO}^4}}{-\lambda\Delta_{\rm CO}^2}\right]$	$\sqrt{\frac{-\alpha}{2(\beta_1-\beta_2)}}$	$-\frac{\alpha^2}{4(\beta_1-\beta_2)}-\frac{\lambda^2\Delta_{\rm CO}^4}{4\beta_2}$

TABLE I: Comparison between different extrema of the free energy in Eq. (10). An additional constraint on the parameters arise as a consequence of the square-root function appearing in the solution for θ.

$\Delta_{d_{xy}} = \Delta_2$ order parameters. The combined order parameter,

$$\boldsymbol{\Delta} = \begin{pmatrix} \Delta_1 \\ \Delta_2 \end{pmatrix}, \tag{6}$$

transforms as the E_{2g} irreducible representation (irrep) of the point group. Writing $\Delta_1 = \Delta_0 \cos\theta e^{i\phi_1}$ and $\Delta_2 = \Delta_0 \sin\theta e^{i\phi_2}$ the Landau free-energy expansion to quartic order is

$$\mathcal{F}_{\rm SC} = \alpha\Delta_0^2 + \beta_1\Delta_0^4 - \beta_2\Delta_0^4 \sin^2 2\theta \sin^2\phi\,, \tag{7}$$

where $\phi = \phi_1 - \phi_2$ is the relative phase between the two order parameters. From here we see immediately the well-known result that for $\beta_2 > 0$ a relative phase of $\phi = \pm\pi/2$ is preferred, whereas for $\beta_2 < 0$, a relative phase of $\phi = 0, \pi$ is selected [18]. Hereafter we will focus on the $\beta_2 > 0$ case and take $\beta_1 > \beta_2$ in order for the free energy to be bounded. In this situation, the $\phi = \pm\pi/2$ phase is selected, implying time-reversal symmetry breaking and a nodeless pairing state.

We now consider what happens inside the charge-ordered (CO) state. Some of the proposed $2 \times 2 \times 2$ charge-order configurations, such as the tri-hexagonal, Star of David, and superimposed tri-hexagonal Star of David phases [19], are triple-$\mathbf{Q}_M$/triple-$\mathbf{Q}_L$ states that preserve the D_{6h} point group of the kagome lattice. Here, $\mathbf{Q}_M$ and $\mathbf{Q}_L$ refer to the wave-vectors $(\frac{1}{2}, \frac{1}{2}, 0)$ and $(\frac{1}{2}, \frac{1}{2}, \frac{1}{2})$ of the Brillouin zone. In these cases, because $\boldsymbol{\Delta}$ continues to transform as the two-dimensional E_{2g} irrep, the superconducting state is expected to remain chiral and nodeless.

However, other proposed $2 \times 2 \times 2$ CDW phases break the threefold rotational symmetry of the lattice, implying that Δ_1 and Δ_2 no longer onset at the same temperature. This is

the case of the so-called staggered tri-hexagonal and staggered Star of David phases [19], which are double-$\mathbf{Q}_L$/single-$\mathbf{Q}_M$ states. In this case, a composite quantity transforming as the E_{2g} irrep of the point group can be constructed from the order parameters of the CO state:

$$\begin{pmatrix} M_1^2 + M_3^2 - 2M_2^2 \\ \sqrt{3}(M_3^2 - M_1^2) \end{pmatrix} , \tag{8}$$

where M_i, with $i = 1, 2, 3$, denote the CO order parameter associated with each wave-vector in the star of $\mathbf{Q}_M$. While $\boldsymbol{\Delta}$ above also transforms as E_{2g}, it cannot be combined with the above composite in the free energy, as it is not gauge-invariant. Nevertheless, we can construct a composite superconducting order parameter combination that is gauge invariant and still transforms as the E_{2g} irrep:

$$\begin{pmatrix} |\Delta_1|^2 - |\Delta_2|^2 \\ -\Delta_1\Delta_2^* - \Delta_1^*\Delta_2 \end{pmatrix} , \tag{9}$$

The "scalar product" between the composites (8) and (9) is now gauge-invariant and transforms trivially under the point group operations and, thus, an allowed term in the free energy expansion. Since we are interested in the fate of the superconducting state inside the CO state, we consider for concreteness and without loss of generality, the particular configuration $M_1 = M_3 = \Delta_{\rm CO}/\sqrt{2}$, $M_2 = 0$. The full expression for the free energy then reads

$$\begin{aligned} \mathcal{F} &= \alpha\Delta_0^2 + \beta_1\Delta_0^4 - \beta_2\Delta_0^4 \sin^2 2\theta \sin^2\phi \\ &\quad + \lambda\Delta_0^2\Delta_{\rm CO}^2 \cos 2\theta \,, \end{aligned} \tag{10}$$

where λ is a coupling constant, assumed hereafter to be positive. Minimization of the free energy yield two possible minima, whose free energies are given by:

$$\mathcal{F}_1 = -\frac{(\alpha - \lambda\eta_1)^2}{4\beta_1} \tag{11}$$

$$\mathcal{F}_2 = -\frac{\alpha^2}{4(\beta_1 - \beta_2)} - \frac{\lambda^2\eta_1^2}{4\beta_2} \,. \tag{12}$$

As summarized in Table I, solution 1 corresponds to a superconducting state where only Δ_1 is non-zero, resulting in a nodal state, since the gap Δ_1 must vanish along the $k_x = \pm k_y$ directions. In contrast, in solution 2, both Δ_1 and Δ_2 are non-zero. Although they have

different magnitudes and their relative phase is no longer $\pm\pi/2$, the total gap function is always finite, implying that solution 2 it is a nodeless state.

The solution that minimizes the free energy depends on the values of α and $\Delta_{\rm CO}^2$. The nodeless state (solution 2) takes place as long as the following condition is met:

$$\Delta_{\rm CO}^2 \leq -\frac{\alpha\beta_2}{\lambda(\beta_1-\beta_2)}\,, \tag{13}$$

which arises from enforcing the argument of square root in the expression for the angle θ_* to be positive (see Table I). When the constraint is saturated, i.e. for

$$\Delta_{\rm CO}^2 = -\frac{\alpha\beta_2}{\lambda(\beta_1-\beta_2)}\,, \tag{14}$$

we have $\mathcal{F}_1 = \mathcal{F}_2$. For larger values of $\Delta_{\rm CO}$, the nodal state (solution 1) is favored. Defining

$$\alpha = \alpha_0(t-1)\,, \tag{15}$$

where $t = \frac{T}{T_{c,0}}$ is the reduced temperature, we can obtain the transition temperatures for both solutions as a function of $\Delta_{\rm CO}^2$. We find

$$t_{c,\rm nodal} = 1 + \frac{\lambda\Delta_{\rm CO}^2}{\alpha_0} \tag{16}$$

$$t_{c,\rm nodeless} = 1 - \frac{\lambda\Delta_{\rm CO}^2}{\alpha_0\beta_2}(\beta_1-\beta_2)\,. \tag{17}$$

Hence, for a finite CO order parameter, the nodal state onsets first, followed by a transition at lower temperatures to a nodeless state. Accordingly, for a fixed temperature, there is a nodeless to nodal transition upon increasing $\Delta_{\rm CO}$. This is illustrated in Supplementary Fig. 14, which shows $t_{c,\rm nodal}$ and $t_{c,\rm nodeless}$ as a function of $\Delta_{\rm CO}$. The specific parameters used in making the figure were: $\alpha_0 = 0.1$, $\beta_1 = 1$, $\beta_2 = 0.4$, and $\lambda = 0.25$.

It is important to emphasize that, even in the nodeless state, the minimum gap value can be very small. To show that, we consider the full gap function

$$\Delta_{\rm tot} = f_{k_x^2-k_y^2}\Delta_0\cos\theta + f_{k_xk_y}\Delta_0\sin\theta e^{i\phi}\,, \tag{18}$$

where $f_{k_x^2-k_y^2}$ and $f_{k_xk_y}$ are form factors that vanish at $k_x = \pm k_y$ and $k_x = 0$ or $k_y = 0$, respectively. Here, θ and ϕ are functions of $\Delta_{\rm CO}$ and are given in Table I. Evaluating

$$\min\left|\frac{\Delta_{\rm tot}}{\Delta_0}\right|\,, \tag{19}$$

as a function of Δ_{CO} gives the inset of Supplementary Fig.14, which was obtained using the same parameters as above and setting the reduced temperature to $t = 0.4$. As expected, the gap minimum vanishes continuously across the nodeless to nodal transition.

The role of the unconventional charge order in the emergence of these unusual superconducting features remained unclear, since the former onsets at a much higher temperature than the latter. This motivated us to explore the superconducting gap structure in both KV_3Sb_5 and RbV_3Sb_5 in the presence and in the absence (total or partial) of charge order, using the disorder-free tuning knob hydrostatic pressure. Our experiments, combined with the theoretical model, shows for the first time that charge order can strongly influence the superconducting gap structure and that a nodal gap is stabilized for a sufficiently large charge order parameter, as we show in the supplementary material. So, depending on the fine details of the charge ordered state in AV3Sb5, either nodal or nodeless state can be found. This can be reason why at ambient pressure we see nodeless SC gap for CsV_3Sb_5 [20] and nodal SC pairing for RbV_3Sb_5 and KV_3Sb_5. But once charge order is either strongly suppressed or fully suppressed, the nodeless state is stabilised for all three compounds, which also spontaneously breaks time-reversal symmetry.

Furthermore, our model shows that the nodal-to-nodeless crossover does not coincide with the full suppression of charge order, unless the transition from the charge-ordered superconducting state to the "pure" superconducting state is first-order. Our phase diagrams suggest that the transition from the charge ordered SC state to the "pure" SC state in KV_3Sb_5 is more likely first-order and more likely second-order like in RbV_3Sb_5. Within the framework of our theoretical model, we expect that the crossover from nodal to nodeless pairing starts at a lower pressure than $p_{\mathrm{co,cr}}$ in RbV_3Sb_5 and at $p_{\mathrm{co,cr}}$ in KV_3Sb_5. This is indeed what is seen experimentally.

---

* Electronic address: `zurab.guguchia@psi.ch`

† Electronic address: `hubertus.luetkens@psi.ch`

‡ Electronic address: rustem.khasanov@psi.ch

[1] Khasanov, R., et. al., High pressure research using muons at the Paul Scherrer Institute. High Pressure Res. **36**, 140-166 (2016).

[2] R. Khasanov, et. al., Pressure-induced electronic phase separation of magnetism and superconductivity in CrAs. *Scientific Reports* **5**, 13788 (2015).

[3] F.O. von Rohr et. al., Unconventional Scaling of the Superfluid Density with the Critical Temperature in Transition Metal Dichalcogenides. Science Advances **5(11)**, eaav8465 (2019).

[4] R. Kubo and T. Toyabe, Magnetic Resonance and Relaxation (North Holland, Amsterdam, 1967).

[5] N.N. Wang, et al. Competition between charge-density-wave and superconductivity in the kagome metal RbV_3Sb_5. Phys. Rev. Research **3**, 043018 (2021).

[6] M. Wenzel, et al., Optical investigations of RbV_3Sb_5: Multiple density-wave gaps and phonon anomalies. Preprint at https://arxiv.org/pdf/2112.07501 (2021).

[7] F. Du, et. al., Pressure-induced double superconducting domes and charge instability in the kagome metal KV_3Sb_5. Phys. Rev. B **103**, L220504 (2021).

[8] Suter, A. and Wojek, B.M. *Physics Procedia* **30**, 69 (2012).
The fitting of the T-dependence of the penetration depth with α model was performed using the additional library BMW developped by B.M. Wojek.

[9] Tinkham, M. Introduction to Superconductivity, *Krieger Publishing Company*, *Malabar*, *Florida*, 1975.

[10] Brandt, E.H. Flux distribution and penetration depth measured by muon spin rotation in high-T_c superconductors. *Phys. Rev. B* **37**, 2349 (1988).

[11] Carrington, A. and Manzano, F. Magnetic penetration depth of MgB_2. *Physica C* **385**, 205 (2003).

[12] Prozorov, R. and Giannetta, R.W. Magnetic penetration depth in unconventional superconductors. *Supercond. Sci. Technol.* **19**, R41 (2006).

[13] Khasanov, et al. Experimental Evidence for Two Gaps in the High-Temperature $La_{1.83}Sr_{0.17}CuO_4$ Superconductor. *Phys. Rev. Lett.* **98**, 057007 (2007).

[14] Khasanov, R. et al. $SrPt_3P$: A two-band single-gap superconductor. *Phys. Rev. B* **90**, 140507(R) (2014).

[15] Kogan, V.G. London approach to anisotropic type-II superconductors. *Phys. Rev. B* **24**, 1572

(1981).

[16] C. Mielke III, et.al., and Z. Guguchia. Time-reversal symmetry-breaking charge order in a kagome superconductor. Nature **602**, 245-250 (2022).

[17] V. G. Kogan, C. Martin, and R. Prozorov, Phys. Rev. B **80**, 014507 (2009).

[18] M. Sigrist and K. Ueda. Phenomenological theory of unconventional superconductivity. Rev. Mod. Phys. **63**, 239 (1991).

[19] M.H. Christensen, et. al., Theory of the charge-density wave in AV_3Sb_5 kagome metals. Phys. Rev. B **104**, 214513 (2021).

[20] R. Gupta et. al., Microscopic evidence for anisotropic multigap superconductivity in the CsV_3Sb_5 kagome superconductor. npj Quantum Materials **7**, 49 (2022).

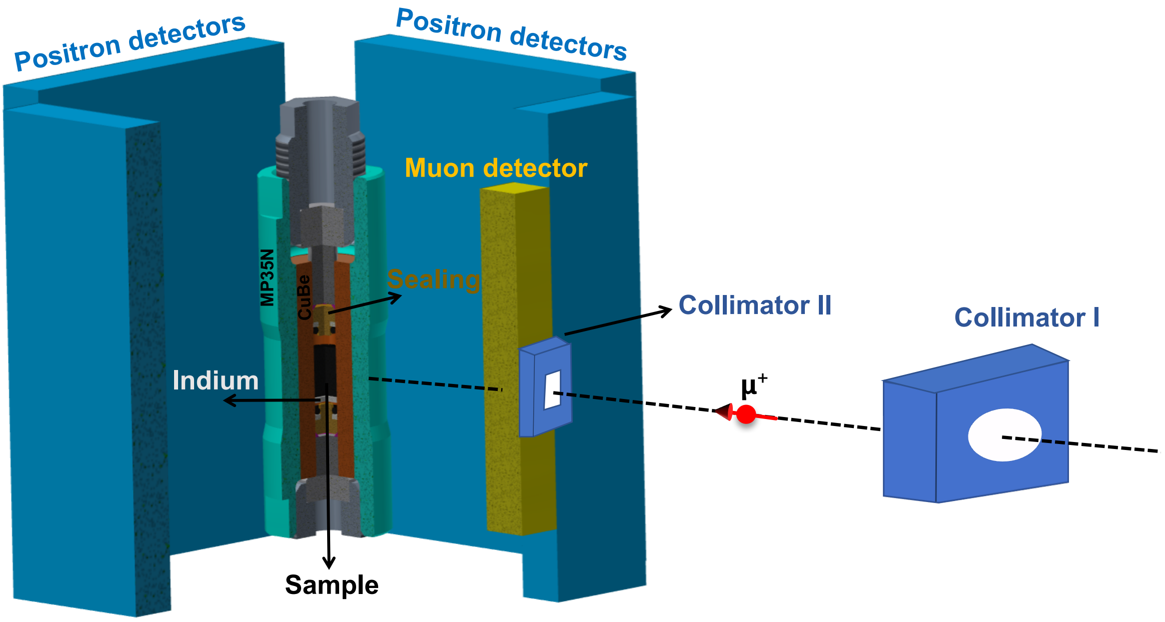

Supplementary Figure 1: **Pressure cell for μSR.** Fully assembled typical double-wall piston-cylinder type of pressure cell used in our μSR experiments. The schematic view of the positron and muon detectors at the GPD spectrometer are also shown. In reality, each positron detector consists of three segments. The collimators reduce the size of the incoming muon beam.

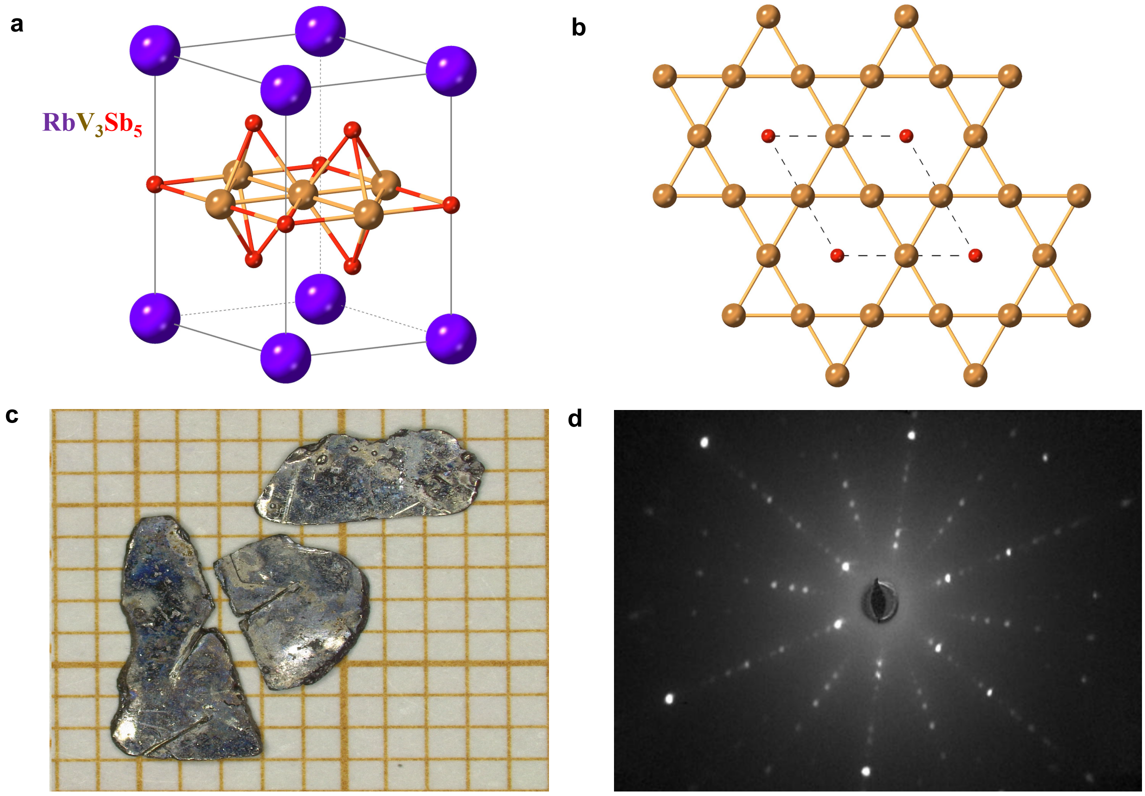

Supplementary Figure 2: **Crystal structure of $\mathbf{RbV_3Sb_5}$.** Three dimensional representation (a) and top view (b) of the atomic structure of RbV_3Sb_5. (c) An optical microscope images of several single crystals of RbV_3Sb_5 on millimeter paper. The hexagonal symmetry is immediately apparent. (d) Laue X-ray diffraction image of the single crystal sample of RbV_3Sb_5, oriented with the *c*-axis along the beam.

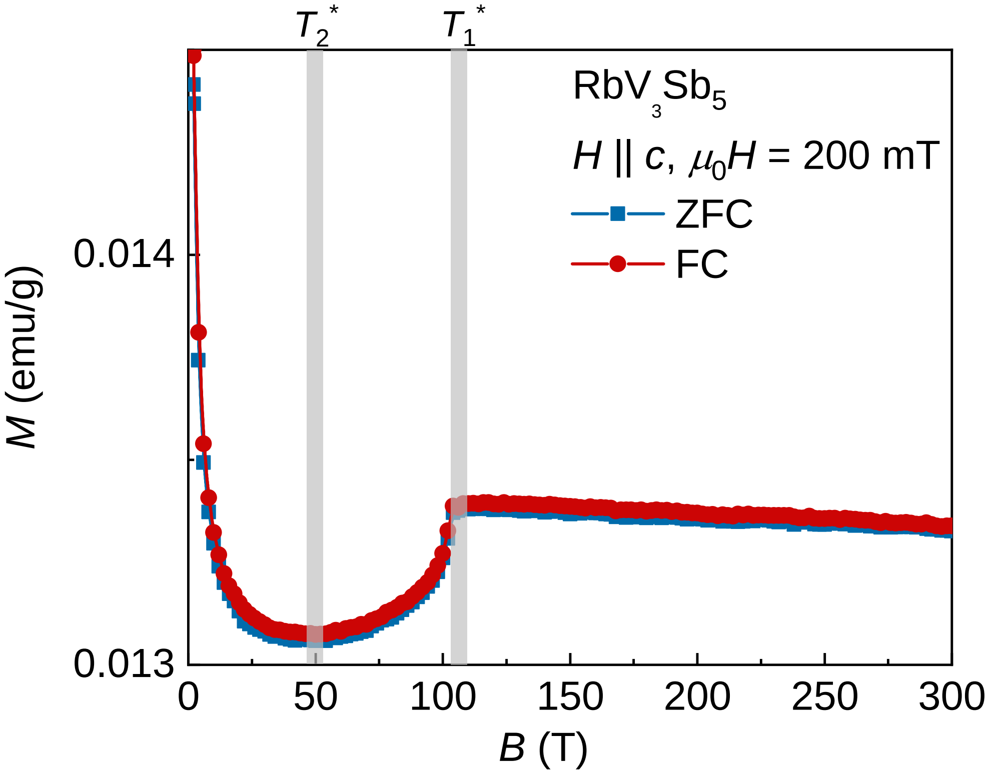

Supplementary Figure 3: **Bulk magnetization for RbV_3Sb_5.** The temperature dependence of magnetic susceptibility of RbV_3Sb_5 above 1.8 K. The vertical grey lines mark the concomitant time-reversal symmetry-breaking and charge ordering temperatures $T_1^* = T_{\mathrm{CDW},1} \simeq 110$ K, $T_2^* = T_{\mathrm{CDW},2} \simeq 50$ K.

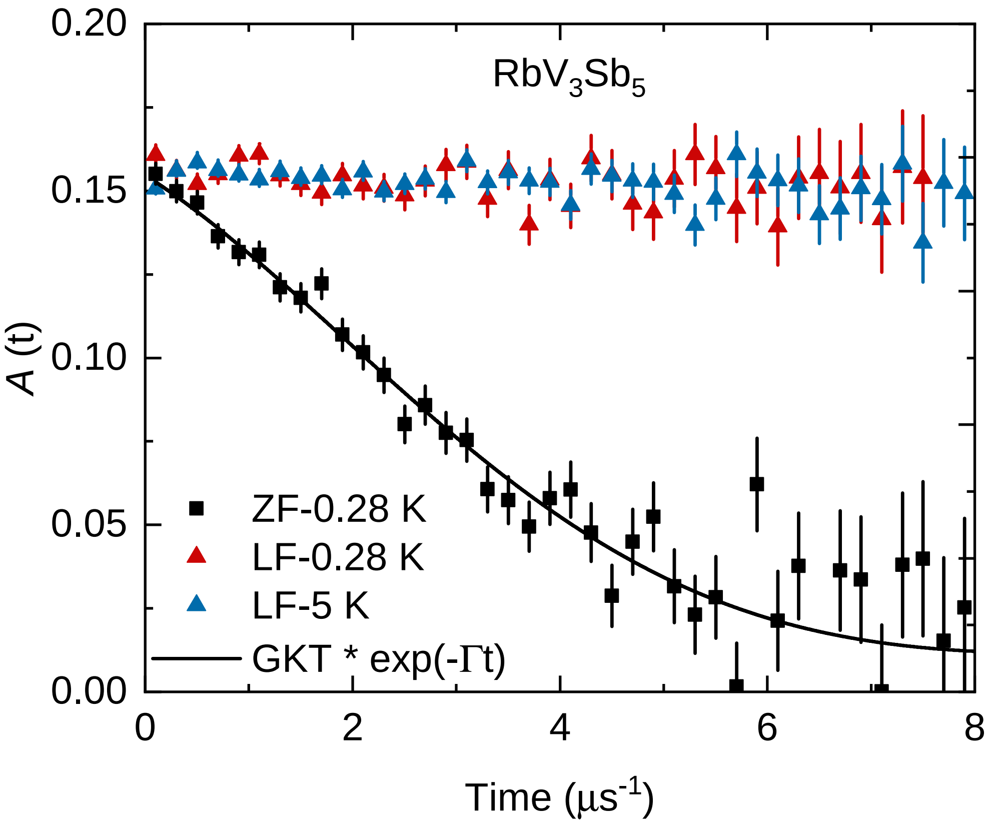

Supplementary Figure 4: **Static nature of internal fields.** The ZF-μSR time spectra for RbV_3Sb_5, obtained at 0.28 K. Longitudinally-applied field (LF) of 50 G clearly decouples the signal both at 0.28 K (below T_c) and at 5 K (above T_c).

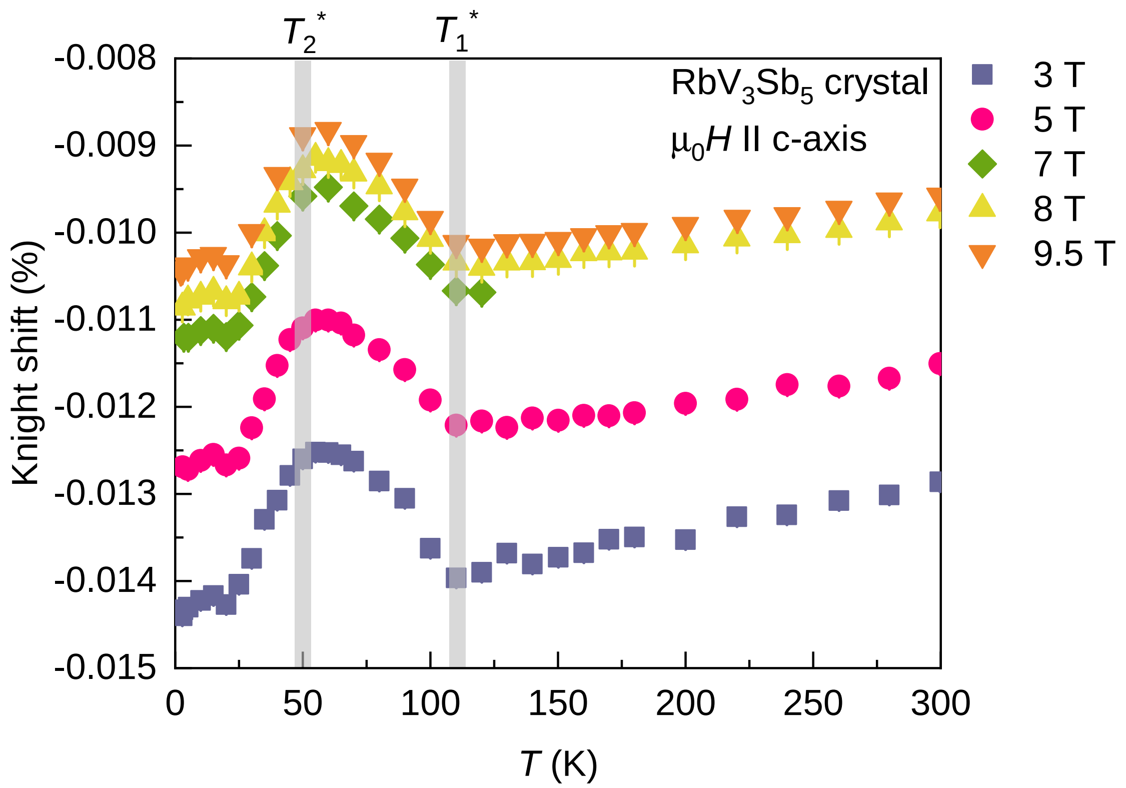

Supplementary Figure 5: **Knight shift for RbV$_3$Sb$_5$.** The temperature dependence of the Knight shift for the single crystal of RbV$_3$Sb$_5$, measured at various magnetic fields applied along the c-axis.

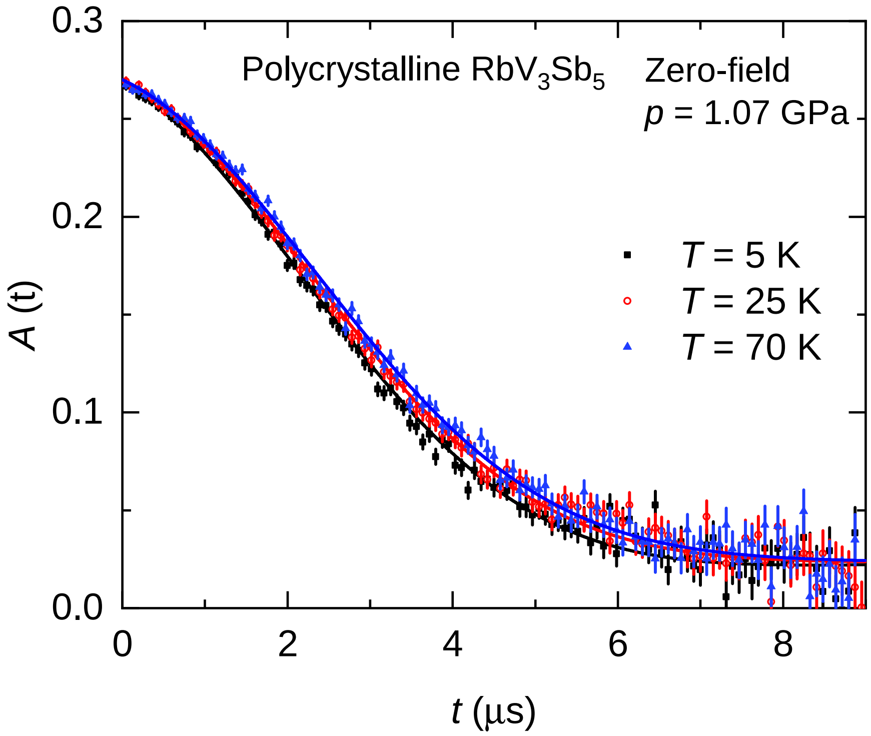

Supplementary Figure 6: **Zero-field spectra of RbV_3Sb_5 under pressure.** The ZF-μSR time spectra for the polycrystalline sample of RbV_3Sb_5, recorded at various temperatures under the applied pressure of $p = 1.07$ GPa. The solid lines in panel a represent fits to the data by means of Eq. 3.

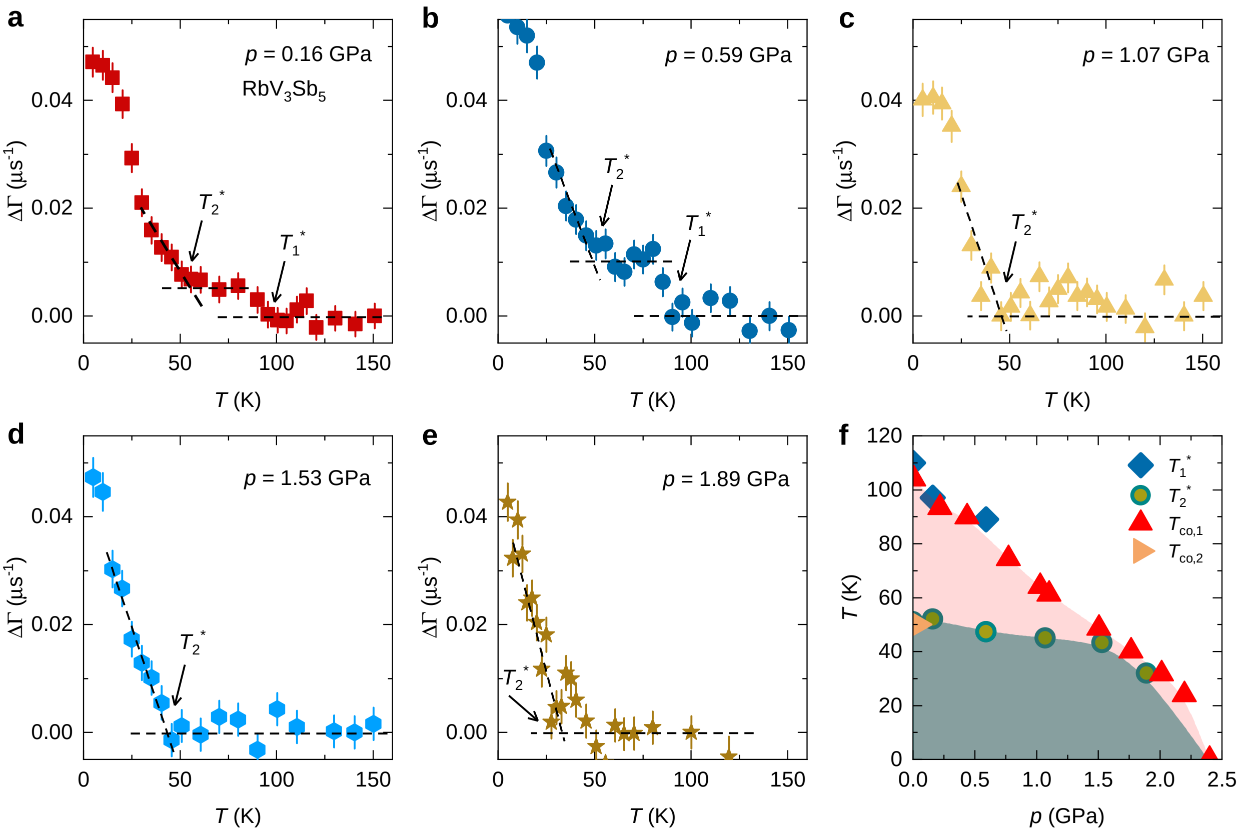

Supplementary Figure 7: (Color online) **Pressure evolution of time-reversal symmetry-breaking charge orders in RbV_3Sb_5.** (a-e) The temperature dependence of the absolute change of the electronic relaxation rate $\Delta\Gamma = \Gamma(T)$ - $\Gamma(T > 150$ K) for the polycrystalline sample of RbV_3Sb_5, measured at various pressures. (f) The charge order temperatures $T_{co,1}$, $T_{co,2}$ (after References [5], [6], [7]) and the onset temperatures of the time-reversal symmetry-breaking T_1^*, T_2^* as a function of pressure.

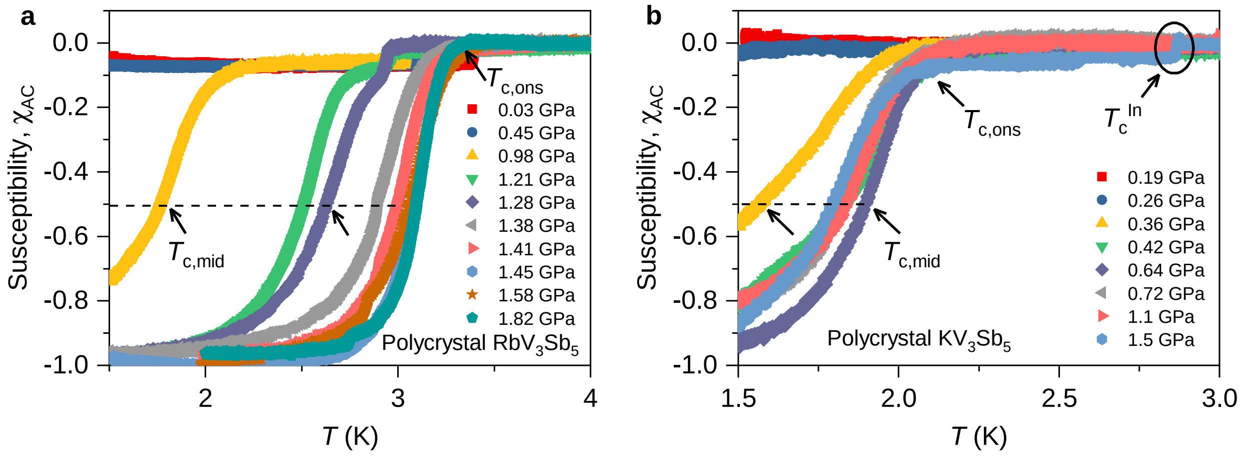

Supplementary Figure 8: **Macroscopic superconducting properties under pressure.** Temperature dependence of the AC susceptibility χ for the polycrystalline samples of RbV_3Sb_5 (a) and KV_3Sb_5 (b), measured at nearly ambient and various applied hydrostatic pressures up to $p \simeq 1.8$ GPa. Arrows mark the onset temperature $T_{c,ons}$ and the temperature $T_{c,mid}$ at which χ_{dc} = -0.5.

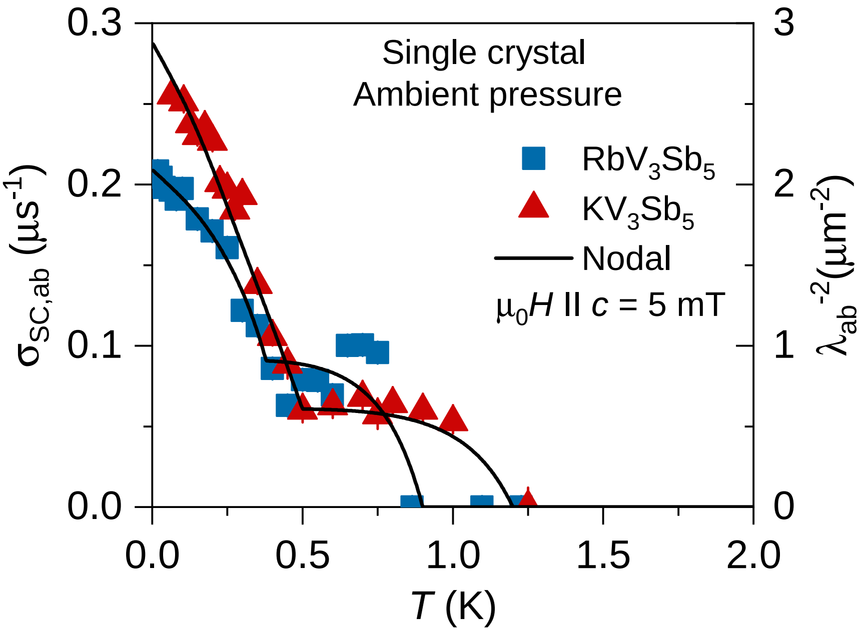

Supplementary Figure 9: **Temperature dependence of the penetration depth at ambient pressure.** The superconducting muon depolarization rate $\sigma_{sc,ab}$ for the single crystals of RbV_3Sb_5 and KV_3Sb_5 as a function of temperature, measured in 5 mT applied perpendicular to the kagome plane. The solid lines correspond to a model with one constant gap and one dominant angle-dependent gap.

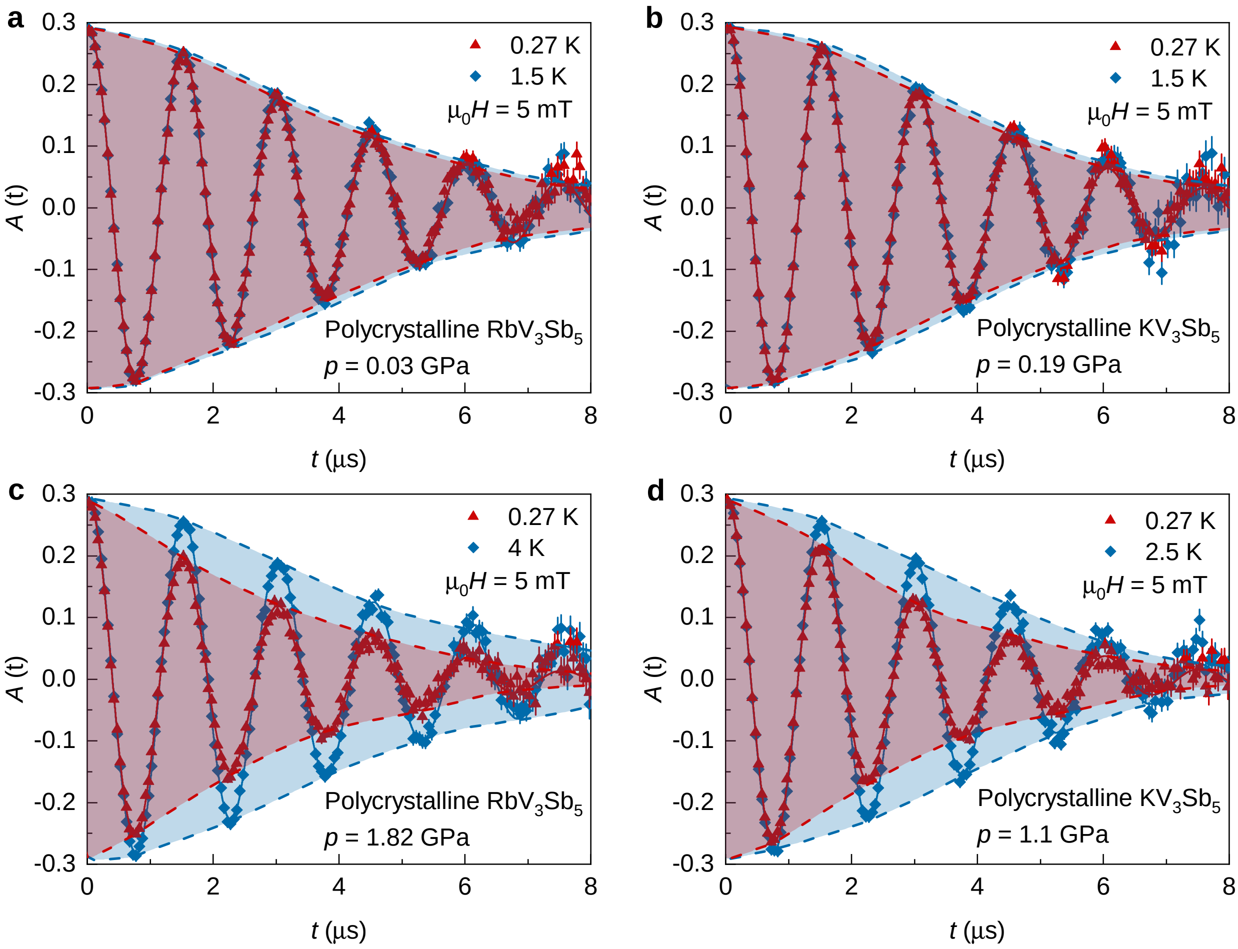

Supplementary Figure 10: **Comparison of superconducting gap models.** The transverse field μSR spectra for RbV_3Sb_5 (a,c) and KV_3Sb_5 (b,d), obtained above and below T_c (after field cooling the sample from above T_c) close to ambient pressure (a and b) and at the maximum applied pressure (c and d). Error bars are the standard error of the mean (s.e.m.) in about 10^6 events. The error of each bin count n is given by the standard deviation (s.d.) of n. The errors of each bin in $A(t)$ are then calculated by s.e. propagation. The solid lines in panel a represent fits to the data by means of Eq. 5. The dashed lines are the guides to the eyes.

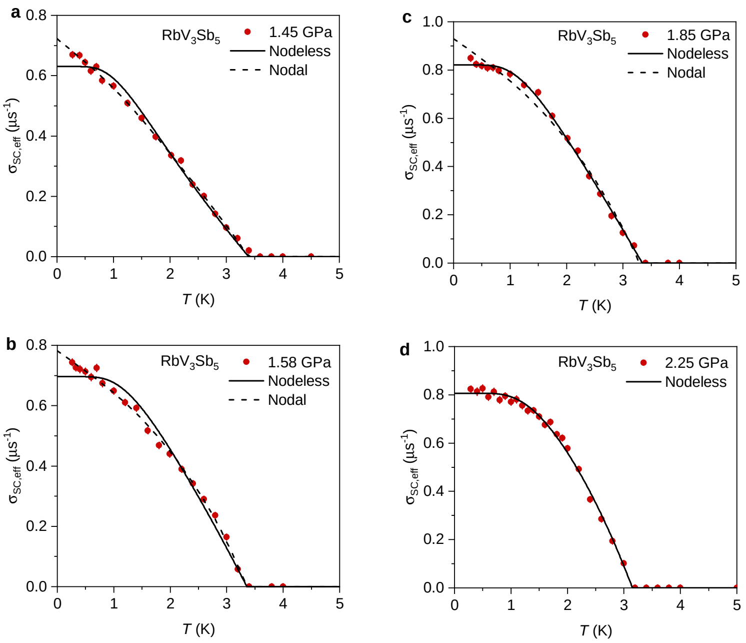

Supplementary Figure 11: **Comparison of superconducting gap models in RbV_3Sb_5.** The temperature dependence of the superconducting muon spin depolarization rates $\sigma_{\rm sc}$ for RbV_3Sb_5, measured in an applied magnetic field of $\mu_0 H = 5$ mT at the applied pressure of $p = 1.45$ GPa (a), $p = 1.58$ GPa (b), and $p = 1.85$ GPa (c). The error bars represent the standard deviations of the fit parameters. The solid (dashed) lines correspond to a fit using a model with nodeless (nodal) gap superconductivity.

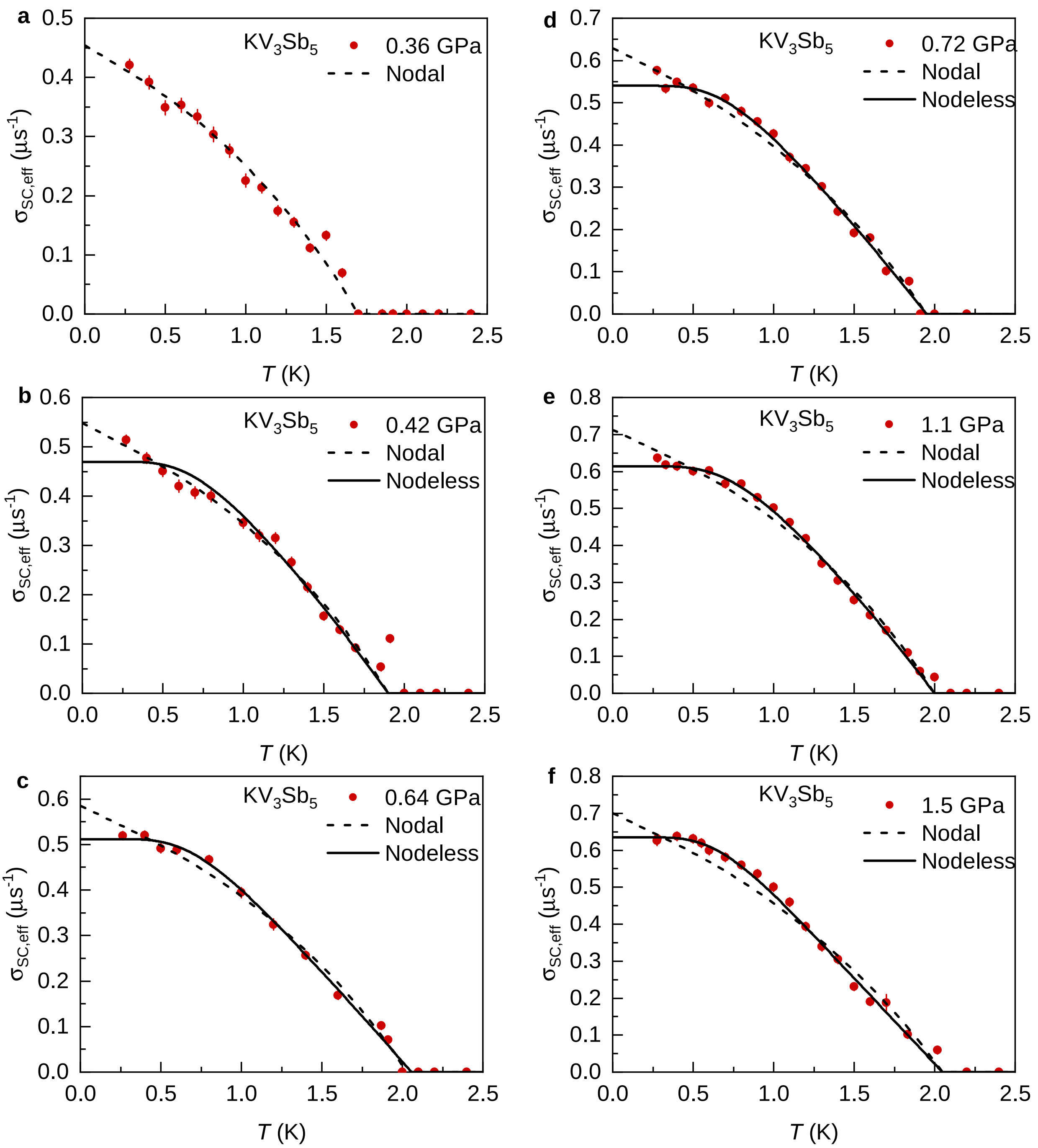

Supplementary Figure 12: **Comparison of superconducting gap models in KV_3Sb_5.** The temperature dependence of the superconducting muon spin depolarization rates σ_{sc} for KV_3Sb_5, measured in an applied magnetic field of $\mu_0 H = 5$ mT at the applied pressure of $p = 0.36$ GPa (a), $p = 0.42$ GPa (b), $p = 0.64$ GPa (c), $p = 0.72$ GPa (d), $p = 1.1$ GPa (e), $p = 1.5$ GPa (f). The error bars represent the standard deviations of the fit parameters. The solid (dashed) lines correspond to a fit using a model with nodeless (nodal) gap superconductivity.

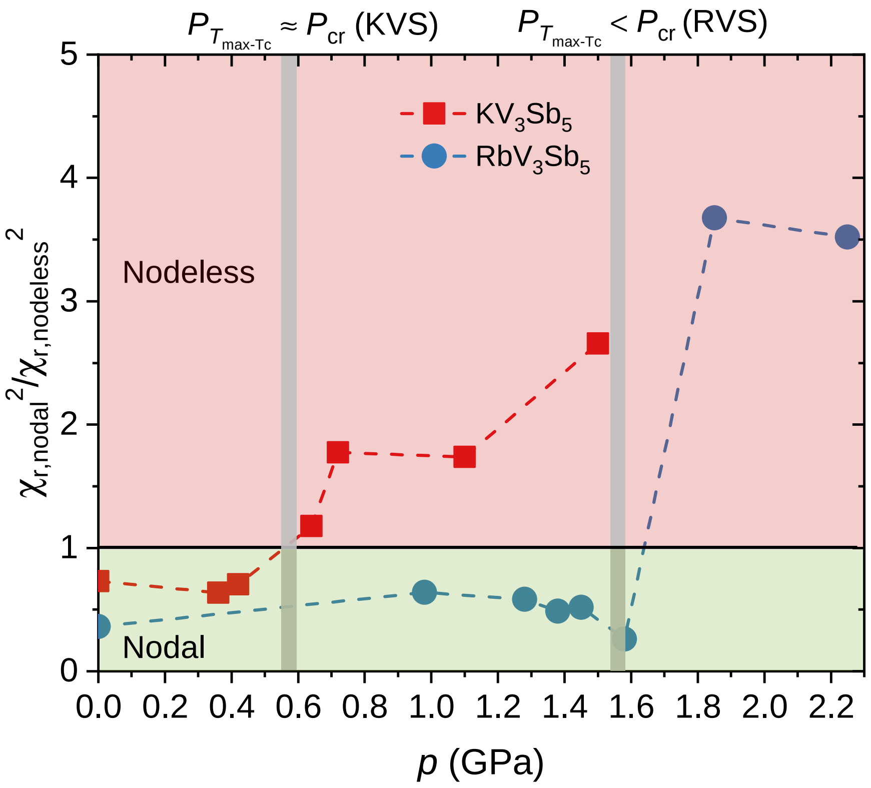

Supplementary Figure 13: $\chi_r{}^2$ **vs pressure.** Pressure dependence of the ratio between the $\chi_r{}^2$ values of nodal and nodeless gap models.

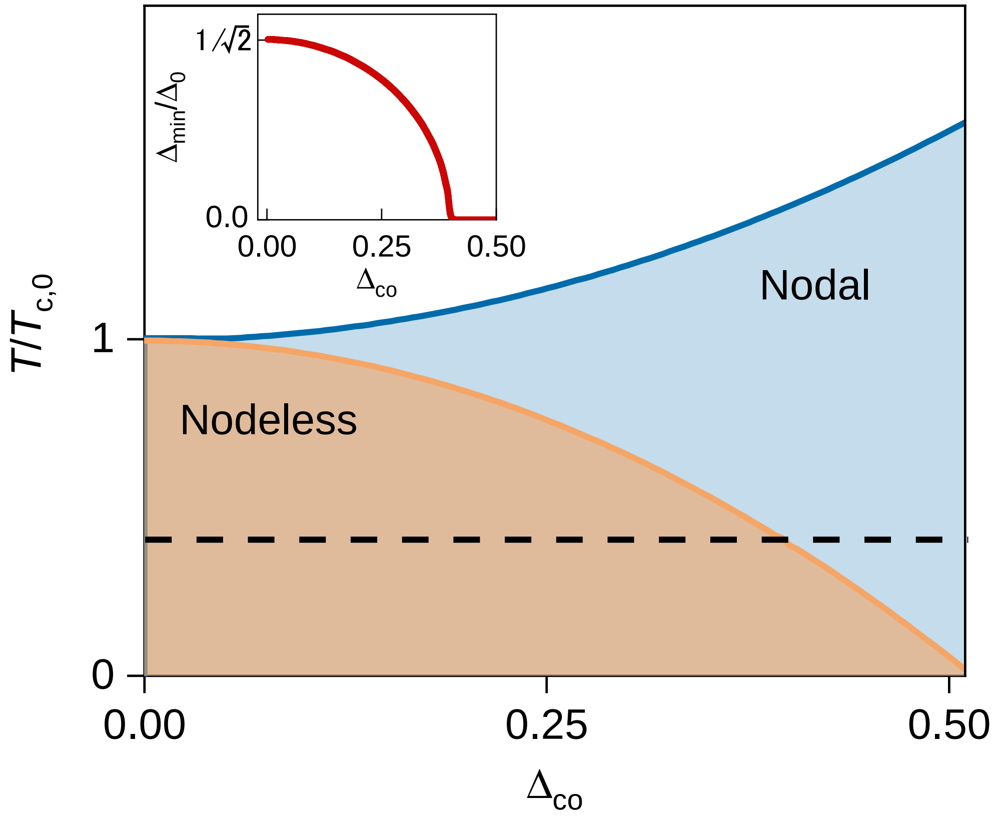

Supplementary Figure 14: (Color online) **Calculated superconducting phase diagram.** Normalized superconducting critical temperature as a function of the charge order parameter, Δ_{co}. As Δ_{co} is increased, a transition from nodeless to nodal superconductivity occurs. As evidenced in Fig. **??c** and **d**, the charge order is suppressed by pressure. As pressure is increased, Δ_{co} is reduced, and the superconducting state goes from nodal to nodeless. The inset shows the minimum gap magnitude as a function of Δ_{co} plotted along the dashed line in the phase diagram. The transition between nodal and nodeless superconductivity is clearly visible.
